# Mutual communication between radiosensitive and radioresistant esophageal cancer cells modulates their radiosensitivity

**DOI:** 10.1038/s41419-023-06307-9

**Published:** 2023-12-19

**Authors:** Congying Xie, Xiao Chen, Yueming Chen, Xingyue Wang, Jiwei Zuo, Anqi Zheng, Zhicheng Luo, Xiaoxiao Cheng, Shouhui Zhong, Jiayu Jiang, Jizao Du, Yuemei Zhao, Peipei Jiang, Wei Zhang, Didi Chen, Huanle Pan, Lanxiao Shen, Baoling Zhu, Qingyu Zhou, Yunsheng Xu, Kai-Fu Tang

**Affiliations:** 1 Zhejiang Engineering Research Center for Innovation and Application of Intelligent Radiotherapy Technology, The Second Affiliated Hospital of Wenzhou Medical University, 325000 Wenzhou, Zhejiang P. R. China; 2 Wenzhou key Laboratory of basic science and translational research of radiation oncology, 325000 Wenzhou, Zhejiang P. R. China; 3 Key Laboratory of Precision Diagnosis and Treatment for Hepatobiliary and Pancreatic Tumor of Zhejiang Province, The Second Affiliated Hospital, Zhejiang University School of Medicine, 310009 Hangzhou, Zhejiang P. R. China; 4 Key Laboratory of Diagnosis and Treatment of Severe Hepato-Pancreatic Diseases of Zhejiang Province, The First Affiliated Hospital of Wenzhou Medical University, 325015 Wenzhou, Zhejiang P. R. China; 5 Department of Breast Surgery, The First Affiliated Hospital of Wenzhou Medical University, 325015 Wenzhou, Zhejiang P. R. China; 6 Department of Radiation Oncology, The First Affiliated Hospital of Wenzhou Medical University, 325015 wenzhou, Zhejiang P. R. China; 7 Department of Radiotherapy Center, The First Affiliated Hospital of Wenzhou Medical University, 325015 Wenzhou, Zhejiang P. R. China; 8 Department of Dermatovenereology, The Seventh Affiliated Hospital, Sun Yat-sen University, 518107 Shenzhen, P. R. China; 9 Key Laboratory of Molecular Biology on Infectious Diseases, Ministry of Education, Chongqing Medical University, 400016 Chongqing, Chongqing P. R. China

**Keywords:** Tumour heterogeneity, Oesophageal cancer, Cancer therapeutic resistance, Radiotherapy, DNA damage response

## Abstract

Radiotherapy is an important treatment modality for patients with esophageal cancer; however, the response to radiation varies among different tumor subpopulations due to tumor heterogeneity. Cancer cells that survive radiotherapy (i.e., radioresistant) may proliferate, ultimately resulting in cancer relapse. However, the interaction between radiosensitive and radioresistant cancer cells remains to be elucidated. In this study, we found that the mutual communication between radiosensitive and radioresistant esophageal cancer cells modulated their radiosensitivity. Radiosensitive cells secreted more exosomal *let-7a* and less interleukin-6 (IL-6) than radioresistant cells. Exosomal *let-7a* secreted by radiosensitive cells increased the radiosensitivity of radioresistant cells, whereas IL-6 secreted by radioresistant cells decreased the radiosensitivity of radiosensitive cells. Although the serum levels of *let-7a* and IL-6 before radiotherapy did not vary significantly between patients with radioresistant and radiosensitive diseases, radiotherapy induced a more pronounced decrease in serum *let-7a* levels and a greater increase in serum IL-6 levels in patients with radioresistant cancer compared to those with radiosensitive cancer. The percentage decrease in serum *let-7a* and the percentage increase in serum IL-6 levels at the early stage of radiotherapy were inversely associated with tumor regression after radiotherapy. Our findings suggest that early changes in serum *let-7a* and IL-6 levels may be used as a biomarker to predict the response to radiotherapy in patients with esophageal cancer and provide new insights into subsequent treatments.

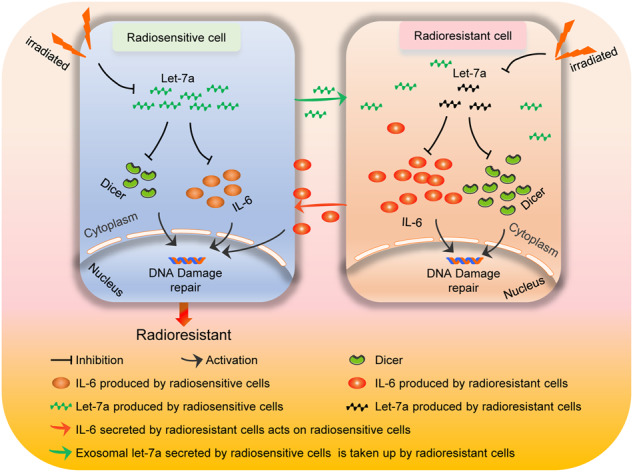

## Introduction

Esophageal cancer is the seventh most common cancer and the sixth most common cause of cancer-related death worldwide [1]. Radiotherapy has become an important treatment option for patients with this disease; however, resistance leads to treatment failure and cancer relapse [2]. DNA damage is the central mediator of the effects elicited by radiotherapy. In particular, ionizing radiation induces DNA damage by causing direct high-energy damage to the sugar backbone of the DNA molecule and by generating free radicals in cells [3]. Moreover, inhibition of DNA repair, as mediated by DNA damage response inhibitors, increases the sensitivity of cancer cells to radiotherapy, whereas an increase in DNA repair activity causes radioresistance [2, 4].

Bulk tumors consist of cancer cells harboring distinct genetic and epigenetic molecular signatures with different levels of sensitivity to anticancer therapies. This heterogeneity results in either a non-uniform distribution of genetically distinct tumor cell subpopulations across, and within, disease sites (i.e., spatial heterogeneity) or temporal variation in the molecular composition of cancer cells (i.e., temporal heterogeneity) [5]. Tumor heterogeneity induces resistance to anticancer therapy. In the simplest scenario, pre-existing resistant cancer cells that survive anticancer treatment may develop and ultimately give rise to resistance and cancer relapse [5].

Cell-cell communication coordinates organismal development, homeostasis, and single-cell functions. In addition to contact-dependent cell-cell communication, the transfer of extracellular vesicles or other secretory factors between cells has been consistently shown to mediate functional communication [6–8]. Cell-cell communication plays pivotal roles in cancer development. For example, at the initial stage of carcinogenesis, normal epithelial cells can recognize the neighboring transformed cells and actively eliminate them from epithelial tissues [9]. Moreover, cancer cells can secrete extracellular vesicles to reprogram stromal cells to support pre-metastatic niche formation and subsequent metastasis [10]. Cell-cell communication is also involved in mediating the effects of radiotherapy. For example, the abscopal effect occurs when radiotherapy at one site leads to the regression of metastatic cancer at distant sites [11]. Evidence accumulated during the last decade has revealed that radiation-induced exosomes contribute to the non-targeted abscopal effect [12]. In addition, radiation-induced exosomes can cause radioresistance [13].

Although our understanding of the role of cell-cell communication in radiosensitivity has progressed, the communication between radiosensitive and radioresistant cancer cells remains to be elucidated. Thus, in this study, we investigated whether radioresistant esophageal cancer cells can decrease the radiosensitivity of radiosensitive cancer cells. Additionally, we sought to determine whether radiosensitive esophageal cancer cells can increase the radiosensitivity of radioresistant cancer cells.

## Results

### Cell–cell communication between radiosensitive and radioresistant esophageal cancer cells mutually modulates their radiosensitivity

**Fig. 1:**
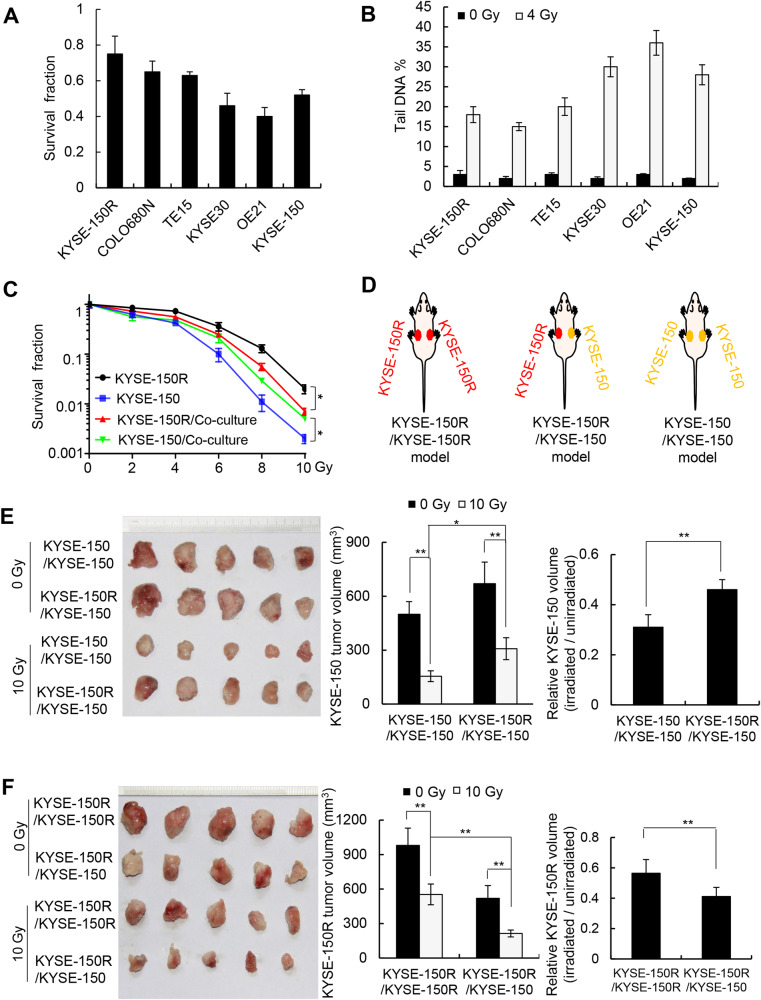
Cell–cell communication between radiosensitive and radioresistant esophageal cancer cells. **A** Survival analysis of KYSE-150R, COLO680N, TE15, KYSE30, OE21, and KYSE-150 cells based on clonogenic assays after 4 Gy of irradiation. **B** DNA breaks in different esophageal squamous cancer cell lines were measured using comet assays 1 h after 4 Gy of irradiation. **C** The radiosensitive KYSE-150 cells alone, the radioresistant KYSE-150R cells alone, the KYSE-150 cells co-cultured with KYSE-150R cells for 48 h, and the KYSE-150R cells co-cultured with KYSE-150 cells for 48 h were exposed to different doses of radiation. Subsequent cell survival was determined using clonogenic assays. Data (A-C) are expressed as the mean ± SD of the values from three independent experiments. **D** Schematic displaying the establishment of the radioresistant (KYSE-150R/KYSE-150R) tumor xenograft mouse model (left), the radioresistant/radiosensitive (KYSE-150R/KYSE-150) xenograft mouse model (middle), and the radiosensitive (KYSE-150/KYSE-150) xenograft mouse model (right). **E** KYSE-150 xenografts on the right flank of the KYSE-150/KYSE-150 mouse model and the KYSE-150R/KYSE-150 mouse model were either exposed to 10 Gy of irradiation or left unirradiated. Xenograft tumors were photographed (left), the average volume was determined (middle), and the volumes of irradiated xenografts relative to those of unirradiated xenografts were calculated (right). **F** KYSE-150R xenografts on the left flank of the KYSE-150R/KYSE-150R mouse model and the KYSE-150R/KYSE-150 mouse model were either exposed to 10 Gy of radiation or left unirradiated. Xenograft tumors were photographed (left), the average volume was determined (middle), and the volumes of irradiated xenografts relative to those of unirradiated xenografts were calculated (right). Data (E and F) are expressed as the mean ± SD of the values obtained from five xenografts. **P < 0.01, *P < 0.05 (two-sided Student’s *t* test).

The radiosensitivity of different esophageal cancer cell lines, including KYSE-150R, COLO680N, TE15, KYSE30, OE21, and KYSE-150 cells, was determined using a clonogenic assay for cell survival and a comet assay for DNA damage. We found that KYSE-150R, COLO680N, and TE15 cells were more resistant to radiation than KYSE30, OE21, and KYSE-150 cells (Fig. 1A, B). To investigate the potential interaction between radiosensitive and radioresistant esophageal cancer cells, radiosensitive and radioresistant cells were co-cultured using a contact-independent co-culture system. We found that co-culture increased radiosensitivity in radioresistant cells and decreased radiosensitivity in radiosensitive cells (Fig. 1C and S1A).

To examine the interaction between radiosensitive and radioresistant cells in vivo, we established three types of tumor xenograft mouse models. For the radiosensitive xenograft mouse model, both the left and right flanks of the nude mouse were subcutaneously inoculated with radiosensitive cells (either KYSE-150 or OE21 cells). For the radioresistant xenograft mouse model, both the left and right flanks of the nude mouse were subcutaneously inoculated with radioresistant cells (either KYSE-150R or COLO680N cells). For the radioresistant/radiosensitive xenograft mouse model, the left and right flanks of the nude mouse were subcutaneously inoculated with radioresistant cells and radiosensitive cells, respectively (Fig. 1D and Fig S1B). As expected, the tumors in the radiosensitive xenograft mouse model displayed a higher sensitivity to irradiation compared to the tumors in the radioresistant xenograft mouse model (Fig S1C). Simultaneous inoculation of radiosensitive and radioresistant cells in the same mouse reduced the radiosensitivity of radiosensitive cells and increased the radiosensitivity of radioresistant cells. Specifically, the radiosensitive xenografts in the radiosensitive/radioresistant xenograft mouse model displayed decreased sensitivity to irradiation compared to those in the radiosensitive xenograft mouse model, whereas the radioresistant xenografts in the radiosensitive/radioresistant xenograft mouse model displayed higher sensitivity to irradiation compared to those in the radioresistant xenograft mouse model (Fig. 1E, F and Fig S1D, E). Taken together, these results indicate that radiosensitive cells can increase the radiosensitivity of radioresistant cells, while radioresistant cells can decrease the radiosensitivity of radiosensitive cells.

### *Let-7a* expression is downregulated in radioresistant esophageal cancer cells

To investigate whether the interaction between radioresistant and radiosensitive esophageal cancer cells is mediated by exosomal miRNAs, we quantified the levels of the top 20 miRNAs highly expressed in esophageal cancer tissues (Table S1) in the culture media of KYSE-150 and KYSE-150R cells. The culture medium of the KYSE-150R cells displayed decreased levels of *miR-21* and *let-7a* and increased levels of *miR-22* when compared with the culture medium of the KYSE-150 cells (Fig S2A).

Moreover, the intracellular levels of *miR-21* and *let-7a* in KYSE-150R cells decreased, while those of *miR-22* increased compared with KYSE-150 cells (Fig S2B). Previous studies demonstrated that the downregulation of *miR-21* expression and the upregulation of *miR-22* expression could sensitize cancer cells to radiotherapy [14–17], excluding the possibility that the downregulation of *miR-21* and the upregulation of *miR-22* can cause radioresistance in KYSE-150R cells. Therefore, in the subsequent analyses, we focused on investigating the role of *let-7a* in regulating the radiosensitivity of esophageal cancer cells.

### Downregulation of *let-7a* induces radioresistance by increasing Dicer expression

**Fig. 2:**
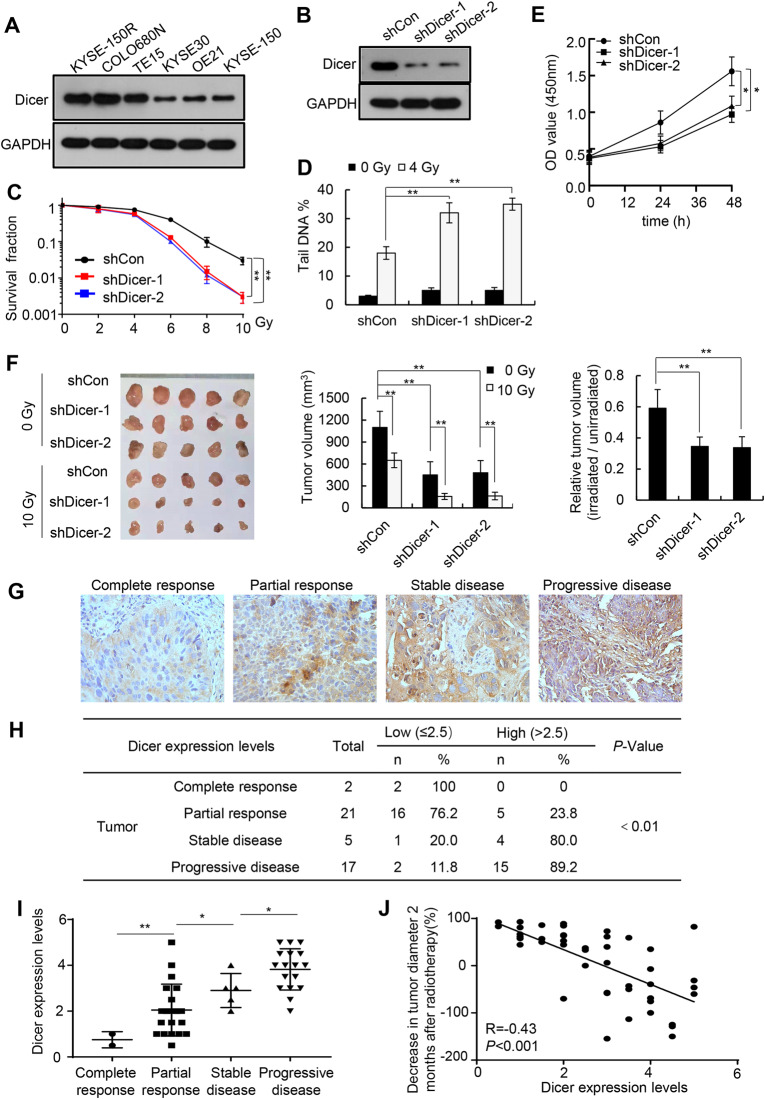
Dicer knockdown promotes esophageal cancer cell sensitivity to radiotherapy. **A** Representative western blotting images of Dicer expression in KYSE-150R, COLO680N, TE15, KYSE30, OE21, and KYSE-150 cells. **B** Representative western blotting images of Dicer expression in the control and Dicer-knockdown KYSE-150R cells. **C** Control and Dicer-knockdown KYSE-150R cells were subjected to different doses of irradiation, and cell survival was determined using clonogenic assays. **D** Control and Dicer-knockdown KYSE-150R cells were subjected to 4 Gy of irradiation, and DNA breaks were determined using comet assays 1 h post-irradiation. **E** The proliferation of control and Dicer-knockdown KYSE-150R cells was determined using CCK-8 assays. Data (**C**–**E**) are expressed as the mean ± SD of values from three biological replicates. **P < 0.01, *P < 0.05 (two-sided Student’s *t* test). **F** Subcutaneous xenografts of either Dicer-knockdown or control KYSE-150R cells were established in athymic nude mice and then subjected to 10 Gy of irradiation. Xenograft tumors were photographed (left), the average volume was determined (middle), and the volumes of irradiated tumors relative to those of unirradiated tumors were calculated (right). Data are expressed as mean ± SD of the values obtained from five xenografts, **P < 0.01 (two-sided Student’s *t* test). **G** Analysis of dicer expression using tumor tissues from patients with esophageal squamous cancer based on immunohistochemistry. Representative images of Dicer expression in cancer tissues from patients with a complete response, a partial response, stable disease, and progressive disease. **H**, **I** Semi-quantitative evaluation of Dicer expression in esophageal cancer tissues. **P < 0.01, *P < 0.05 (two-sided Student’s *t* test). **J** Correlation between Dicer expression and tumor regression (i.e., a decrease in the tumor diameter 2 months after radiotherapy), which was calculated as follows: (the baseline longest diameter of the tumor − the longest diameter of the tumor at 2 months post radiotherapy)/the baseline longest diameter of the tumor × 100%.

Consistent with a previous study [18], dual-luciferase assays confirmed that *let-7a* downregulated the expression of luciferase reporters bearing different fragments of the *Dicer* 3′-UTR in KYSE-150R cells (Fig S2C, D). Transfection of a *let-7a* mimic decreased the Dicer protein levels but not the mRNA levels (Fig S2E–G). Conversely, transfection of a *let-7a* inhibitor increased the Dicer protein levels but not the mRNA levels (Fig S2H–J). Given that Dicer is required for DNA damage repair and that Dicer expression levels in cancer tissues are associated with chemosensitivity in patients with colon cancer [19–21], we speculated that *let-7a* downregulation could induce radioresistance by increasing Dicer expression. To test this hypothesis, we first investigated the regulatory effects of Dicer on the radiosensitivity of human esophageal squamous cancer cells. Dicer expression levels were higher in radioresistant cell lines (KYSE-150R, COLO680N, and TE15) than in radiosensitive cell lines (KYSE30, OE21, and KYSE-150) (Fig. 2A). Furthermore, radiation-induced Dicer expression was observed in human esophageal cancer cells in a dose-dependent manner (Fig S3A). Dicer knockdown in radioresistant KYSE-150R cells increased radiosensitivity and decreased cell proliferation (Fig. 2B–E), whereas its overexpression in radiosensitive KYSE-150 cells reduced radiosensitivity and decreased cell proliferation (Fig S3B–E). Dicer overexpression also reduced the radiosensitivity of the KYSE-150 xenografts (Fig S3F), whereas its knockdown increased the radiosensitivity of the KYSE-150R xenografts (Fig. 2F).

Analysis of Dicer expression in 45 esophageal cancer tissues further revealed that Dicer protein levels in radioresistant esophageal squamous cancer tissues were higher than those in radiosensitive cancer tissues (Fig. 2G–I). Furthermore, the Dicer expression levels were inversely correlated with tumor regression, as assessed by changes in the longest tumor diameter after 2 months of radiotherapy (Fig. 2J). Taken together, these findings indicate that Dicer regulates the radiosensitivity of human esophageal squamous cancer cells.

**Fig. 3:**
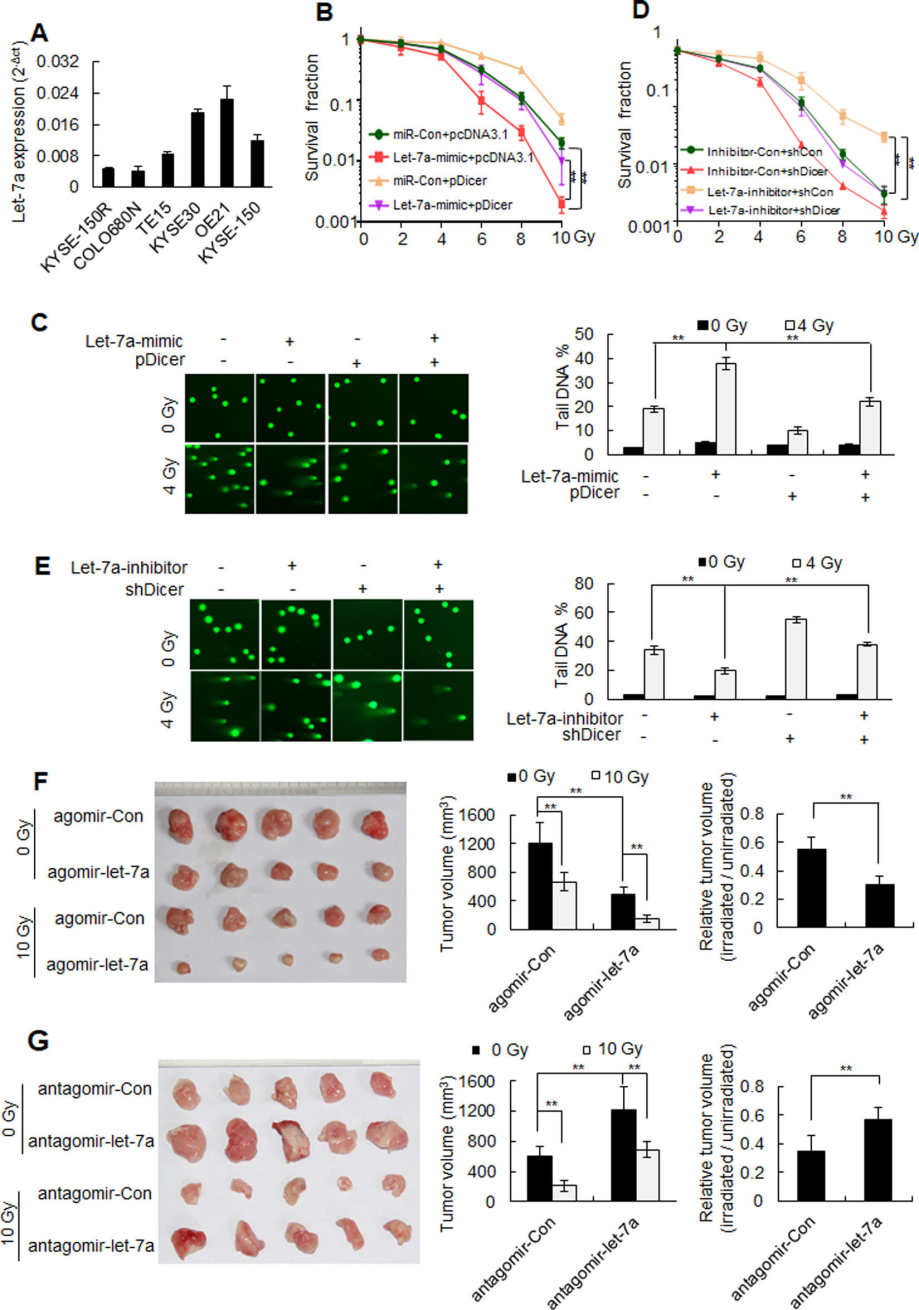
*Let-7a* increases the sensitivity of esophageal cancer cells to radiotherapy by regulating Dicer expression. **A** Real-time RT-PCR quantification of *let-7a* levels in different esophageal squamous cancer cell lines. **B**, **C** KYSE-150R cells were co-transfected with a miR-Con*/let-7a* mimic and pcDNA3.1/pDicer, as indicated, and treated with different doses of radiation 48 h post transfection. Cell survival was determined using clonogenic assays (**B**), and DNA breaks were measured using comet assays (**C**). **D**, **E** KYSE-150 cells were co-transfected with an inhibitor-Con/*let-7a*-inhibitor and shCon/shDicer, as indicated, and treated with different doses of radiation 48 h post transfection. Cell survival was determined using clonogenic assays (D), and DNA breaks were measured using comet assays (**E**). Data (**A**–**E**) are expressed as the mean ± SD of the values from three independent experiments. **P < 0.01 (two-sided Student’s *t* test). **F** Subcutaneous xenografts of KYSE-150R cells were irradiated, followed by intratumoral injection of either agomir-*let-7a* or agomir-Con. Xenograft tumors were photographed (left), the average volume was determined (middle), and the volumes of irradiated tumors relative to those of unirradiated tumors were calculated (right). **G** Subcutaneous xenografts of KYSE-150 cells were irradiated, followed by intratumoral injection of antagomir-*let-7a*. Xenograft tumors were photographed (left), the average volume was determined (middle), and the volumes of irradiated tumors relative to those of unirradiated tumors were calculated (right). Data (**F**, **G**) are expressed as the mean ± SD of five xenografts. **P < 0.01 (two-sided Student’s *t* test).

Upon quantifying the expression of *let-7a* in a set of human esophageal squamous cancer cell lines, we found that *let-7a* expression levels were higher in radiosensitive cells (KYSE30, OE21, and KYSE-150) than in radioresistant cells (KYSE-150R, COLO680N, and TE15) (Fig. 3A). The transfection of *let-7a* mimics increased radiosensitivity in cultured human esophageal cancer cells, which was blocked by ectopic expression of the human Dicer protein (Fig. 3B, C; Fig S4A, B). Moreover, transfection with *let-7a* inhibitors decreased radiosensitivity in these cells, which was blocked by *Dicer* knockdown (Fig. 3D, E; Fig S4C, D). Notably, we found that intratumoral injection of the *let-7a* agomir increased the radiosensitivity of KYSE-150R xenografts (Fig. 3F), whereas intratumoral injection of the *let-7a* antagomir decreased the radiosensitivity of KYSE-150 xenografts (Fig. 3G). Consistent with previous reports [22, 23], we found that *let-7a* overexpression repressed KYSE-150R cell proliferation and KYSE-150R xenograft growth, while *let-7a* inhibition promoted KYSE-150 cell proliferation and KYSE-150 xenograft growth (Fig. 3F, G; S4E, F). Collectively, these findings indicate that *let-7a* downregulation induces radioresistance and promotes tumor growth.

### Exosomal *let-7a* secreted by radiosensitive cells increases the radiosensitivity of radioresistant cells

**Fig. 4:**
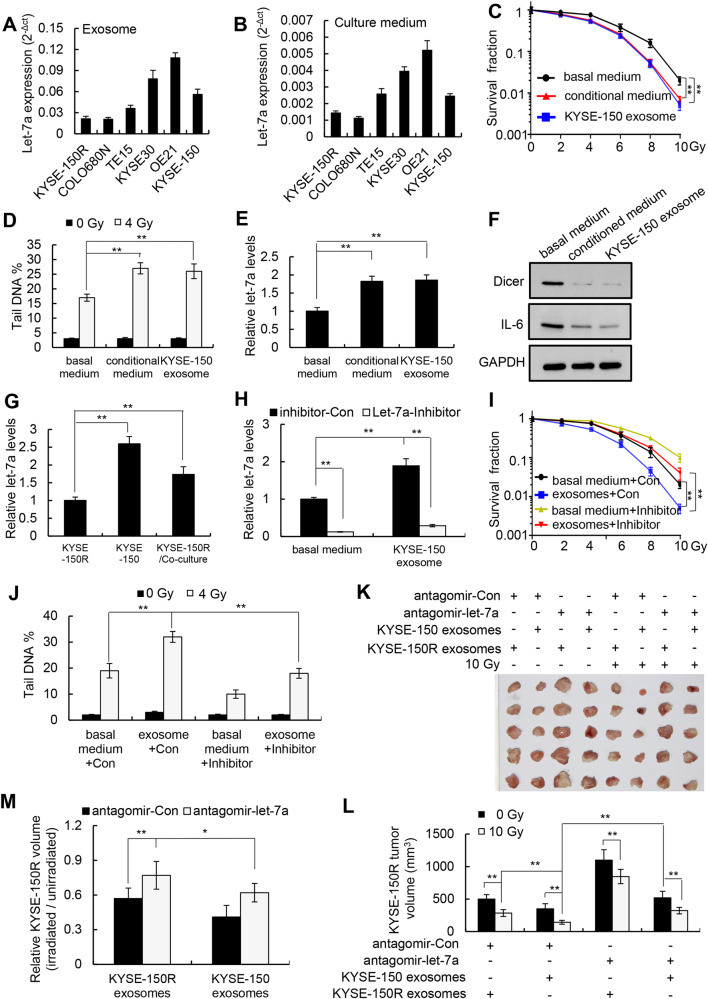
*Let-7a* secreted by KYSE-150 cells increases the sensitivity of KYSE-150R cells to radiotherapy. **A**, **B** Real-time RT-PCR quantification of *let-7a* levels in the exosomes (**A**) and cell culture medium (**B**) of different esophageal squamous cancer cell lines. **C**, **D** KYSE-150R cells were incubated with either basal medium, KYSE-150-conditioned medium (culture medium collected from KYSE-150 cells), or basal medium supplemented with KYSE-150 exosomes for 48 h and exposed to different doses of radiation. Cell survival was determined using clonogenic assays (**C**), and DNA breaks were measured using comet assays (**D**). **E**, **F** KYSE-150R cells were incubated for 48 h with either basal medium, KYSE-150-conditioned medium, or basal medium supplemented with KYSE-150 exosomes. Intracellular levels of *let-7a* (E), Dicer, and IL-6 (F) were determined. **G** Real-time RT-PCR quantification of intracellular *let-7a* levels in KYSE-150R cells, KYSE-150 cells, and KYSE-150R cells co-cultured with KYSE-150 cells for 48 h. **H**–**J** KYSE-150R cells were transfected with either *let-7a* inhibitors or control inhibitors and incubated with either basal medium or basal medium supplemented with KYSE-150 exosomes for 48 h and exposed to different doses of radiation. Intracellular *let-7a* levels were quantified using real-time RT-PCR (H), cell survival was determined using clonogenic assays (I), and DNA breaks were measured using comet assays (**J**). Data (**A**–**E**, **G**–**J**) are expressed as the mean ± SD of the values from three independent experiments. **P < 0.01 (two-sided Student’s *t* test). **K**–**M** Subcutaneous xenografts of KYSE-150R cells were intratumorally injected with either KYSE-150 or KYSE-150R exosomes and exposed to 10 Gy of radiation, followed by an intratumoral injection of antagomir-*let-7a* or antagomir-Con. Xenograft tumors were photographed (**K**), the average volume was determined (**L**), and the volumes of irradiated xenografts relative to those of unirradiated xenografts were measured (**M**). Data (**L**, **M**) are expressed as the mean ± SD of the values obtained from five xenografts. *P < 0.05, **P < 0.01 (two-sided Student’s *t* test).

*Let-7a* levels in the exosomes and culture medium of radiosensitive cells (KYSE30, OE21, and KYSE-150) were higher than those in radioresistant cells (KYSE-150R, COLO680N, and TE15) (Fig. 4A, B). To determine whether exosomal *let-7a* secreted by radiosensitive cells can be transferred to radioresistant cells to increase their radiosensitivity, we purified exosomes from KYSE-150 cells. The quality of the isolated exosomes was confirmed using two well-known exosome markers, CD63 and CD81 [24] (Fig S5A). Incubation with either a conditional medium collected from KYSE-150 cells or a basal medium supplemented with exosomes isolated from KYSE-150 cells (i.e., KYSE-150 exosomes) increased the radiosensitivity and intracellular levels of *let-7a* and decreased the intracellular levels of Dicer and IL-6 in KYSE-150R cells but did not affect the intracellular levels of pri-*let-7a* and pre-*let-7a* (Fig. 4C–F; Fig S5B). In addition, fluorescence confocal microscopy confirmed that PKH67-labeled KYSE-150 exosomes were taken up by KYSE-150R cells (Fig S5C). Co-culture of KYSE-150R cells with radiosensitive KYSE-150 cells also increased the intracellular levels of *let-7a* in KYSE-150R cells without affecting the *pri-let-7a* or *pre-let-7a* levels in these cells (Fig. 4G; Fig S5D). Additionally, we showed that transfection of *let-7a* inhibitors into KYSE-150R cells reversed the KYSE-150 exosome-induced increase in radiosensitivity, indicating that exosomal *let-7a* derived from radiosensitive KYSE-150 cells could increase the radiosensitivity of radioresistant KYSE-150R cells (Fig. 4H–J). Finally, intratumoral injection of exosomes purified from the serum of KYSE-150 xenograft nude mice into KYSE-150R xenografts increased their radiosensitivity, whereas co-injection with the *let-7a* antagomir into KYSE-150R xenografts blocked this effect (Fig. 4K–M). Taken together, these results indicate that radiosensitive KYSE-150 cells can increase the radiosensitivity of radioresistant KYSE-150R cells through the secretion of exosomal *let-7a*.

### IL-6 secreted by radioresistant cells decreases the radiosensitivity of radiosensitive cells

**Fig. 5:**
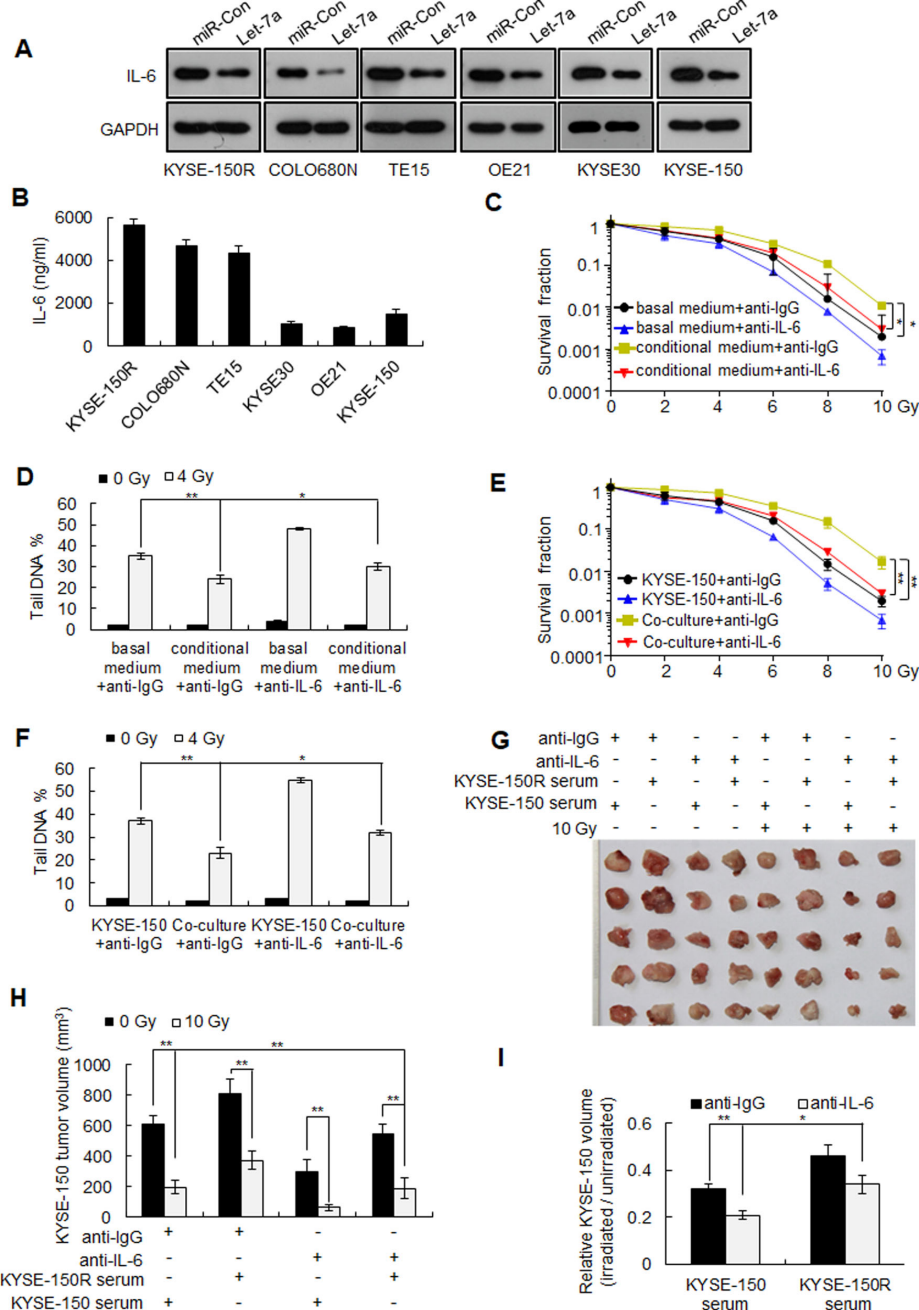
IL-6 secreted by KYSE-150R cells decreases the sensitivity of KYSE-150 cells to radiotherapy. **A** IL-6 protein levels in *let-7a* mimic-transfected human esophageal squamous cancer cell lines (KYSE-150R, COLO680N, TE15, OE21, KYSE30, and KYSE-150) were determined using western blotting. **B** ELISA-based quantification of IL-6 protein levels in the cell culture medium of different esophageal squamous cancer cell lines. **C**, **D** KYSE-150 cells were incubated in either basal medium or KYSE-150R-conditioned medium (culture medium collected from KYSE-150R cells) along with either an anti-IL-6 or IgG control antibody (10 ng/mL) and exposed to different doses of radiation. Cell survival was determined using clonogenic assays (**C**), and DNA breaks were measured using comet assays (**D**). **E**, **F** KYSE-150 cells were co-cultured with KYSE-150R cells in the presence of either the anti-IL-6 or IgG control (10 ng/mL) antibody and exposed to different doses of radiation. KYSE-150 cell survival was determined using clonogenic assays (**E**), and DNA breaks were measured using comet assays (**F**). Data (B-F) are expressed as the mean ± SD of the values from three independent experiments. *P < 0.05, **P < 0.01 (two-sided Student’s *t* test). **G–I** KYSE-150/KYSE-150 model mice were intravenously co-injected with either KYSE-150R serum (serum from mice bearing KYSE-150R xenografts) or KYSE-150 serum (serum from mice bearing KYSE-150 xenografts) and either the anti-IL-6 or IgG control antibody, as indicated, and exposed to 10 Gy of radiation. Xenograft tumors were photographed (**G**), the average volume was determined (**H**), and the volumes of irradiated xenografts relative to those of unirradiated xenografts were calculated (**I**). Data (**H**, **I**) are expressed as the mean ± SD of the values obtained from five xenografts. *P < 0.05, **P < 0.01 (two-sided Student’s *t* test).

Radiotherapy induces IL-6 expression, which in turn promotes DNA repair, thereby causing radioresistance [25]. IL-6 is a target of *let-7a*, which can repress IL-6 expression, either directly or indirectly [26, 27]. However, other studies have reported that *let-7a* promotes IL-6 expression via different mechanisms [28, 29]. We found *let-7a* inhibited IL-6 expression in different human esophageal squamous cancer cell lines (Fig. 5A). Moreover, radioresistant cells (KYSE-150R, COLO680N, and TE15) expressed higher levels of IL-6 compared with radiosensitive cells (KYSE30, OE21, and KYSE-150) (Fig. 5B). Therefore, we investigated whether radioresistant cells could reduce the radiosensitivity of radiosensitive cells via the secretion of IL-6. Incubation with the conditioned medium from the KYSE-150R cells reduced the radiosensitivity of the KYSE-150 cells, but this activity was blocked following the addition of an anti-IL-6 antibody to the culture medium (Fig. 5C, D). Moreover, the addition of an anti-IL-6 antibody to the culture medium prevented the reduction in the radiosensitivity of KYSE-150 cells when co-cultured with radioresistant KYSE-150R cells (Fig. 5E, F). Finally, intravenous injection of serum collected from KYSE-150R xenograft mice decreased the radiosensitivity of KYSE-150 xenografts, whereas co-injecting with anti-IL-6 antibody blocked this effect (Fig. 5G–I). Taken together, these results indicate that radioresistant KYSE-150R cells can decrease the radiosensitivity of radiosensitive KYSE-150 cells via the secretion of IL-6.

### Changes in serum *let-7a* and IL-6 levels are associated with radiosensitivity in esophageal squamous cancer patients

To determine whether the levels of *let-7a* and IL-6 correlate with clinical parameters in patients with esophageal squamous cancer, we analyzed data from 184 patients in the TCGA database. Increased levels of *let-7a* in esophageal squamous cancer tissues were associated with increased median survival (Fig S6A), and increased levels of *IL-6* mRNA were associated with decreased median survival (Fig S6B).

To determine whether the levels of *let-7a* and *IL-6* were correlated with radiosensitivity in patients with esophageal squamous cancer, we compared the levels of *let-7a* and *IL-6* mRNA in the cancer tissues of 31 patients exhibiting a complete response and 13 patients presenting with progressive disease. As shown in Fig S6C and S6D, the levels of *let-7a* and *IL-6* mRNA in the esophageal cancer tissues of patients with a complete response did not differ significantly from those of patients with progressive disease.

**Fig. 6:**
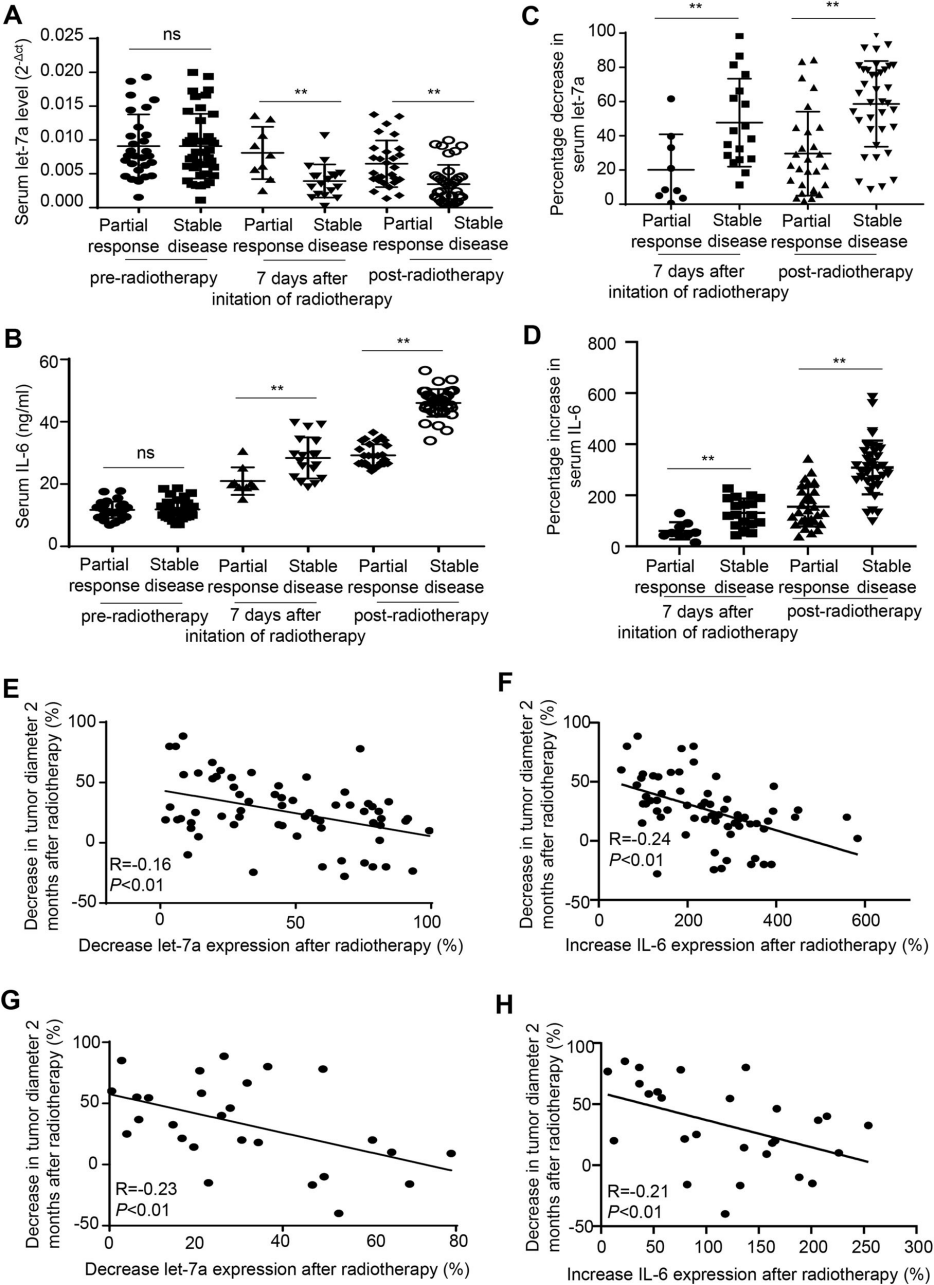
Changes in serum *let-7a* and IL-6 levels are associated with radiosensitivity in esophageal squamous cancer. **A** Pre- and post-radiotherapy serum *let-7a* levels were determined using real-time RT-PCR. **B** Pre- and post-radiotherapy serum IL-6 levels were quantified using ELISA. **C** The percentage decrease in serum *let-7a* levels was determined 7 days following initiation of radiotherapy and after the completion of radiotherapy. The percentage decrease in serum *let-7a* levels was calculated as follows: (pre-radiotherapy serum *let-7a* level − serum *let-7a* levels at 7 days after the initiation of radiotherapy or after the completion of radiotherapy)/pre-radiotherapy serum *let-7a* level × 100%. **D** The percentage increase in serum IL-6 levels was determined 7 days following initiation of radiotherapy and after the completion of radiotherapy. The percentage increase in serum IL-6 levels was calculated as follows: (serum IL-6 levels at day 7 following initiation of radiotherapy or after the completion of radiotherapy—pre-radiotherapy serum IL-6 level)/pre-radiotherapy serum IL-6 level × 100%. *P < 0.05, **P < 0.01, ns, not significant (two-sided Student’s *t* test). **E** The percen*t*age decrease in serum *let-7a* levels induced by 60 Gy of radiotherapy was inversely correlated with tumor regression. The percentage decrease in serum *let-7a* levels induced by 60 Gy of radiotherapy was calculated as follows: (pre-radiotherapy serum *let-7a* level—post-radiotherapy serum *let-7a* level)/pre-radiotherapy serum *let-7a* level × 100%. The percentage decrease in the tumor diameter 2 months post radiotherapy was calculated as follows: (the baseline longest diameter of the tumor—the longest diameter of the tumor at 2 months post radiotherapy)/the baseline longest diameter of the tumor × 100%. **F** The percentage increase in serum IL-6 levels induced by 60 Gy of radiotherapy was inversely correlated with tumor regression. The percentage increase in serum IL-6 levels induced by 60 Gy of radiotherapy was calculated as follows: (post-radiotherapy serum IL-6 level − pre-radiotherapy serum IL-6 level)/pre-radiotherapy serum IL-6 level × 100%. **G** The percentage decrease in serum *let-7a* levels induced by 10 Gy of radiotherapy was inversely correlated with tumor regression. The percentage decrease in serum *let-7a* levels induced by 10 Gy of radiotherapy was calculated as follows: (pre-radiotherapy serum *let-7a* level – serum *let-7a* level after 10 Gy radiotherapy)/pre-radiotherapy serum *let-7a* × 100%. **H** The percentage increase in serum IL-6 levels induced by 10 Gy of radiotherapy was inversely correlated with tumor regression. The percentage increase in serum IL-6 levels induced by 10 Gy of radiotherapy was calculated as follows: (serum IL-6 level after 10 Gy of radiotherapy—re-radiotherapy serum IL-6 level)/pre-radiotherapy serum IL-6 level × 100%.

We then examined whether the serum levels of *let-7a* and IL-6 correlated with radiosensitivity in a cohort of 70 patients with esophageal squamous cancer, of which 28 were with a partial response and 42 with stable disease. Pre-radiotherapy serum samples were collected from all 70 patients, whereas post-radiotherapy (after 60 Gy radiotherapy) serum samples were collected from 28 patients with a partial response and 37 patients with stable disease. Although the serum *let-7a* and IL-6 levels before radiotherapy did not differ significantly between these patients, the levels of post-radiotherapy serum *let-7a* were higher in patients with a partial response than in those with stable disease. Meanwhile, the levels of post-radiotherapy serum IL-6 were lower in patients with a partial response than in those with stable disease (Fig. 6A, B). Interestingly, the effect of radiotherapy-induced alterations in serum *let-7a* and IL-6 levels was more profound in patients with radioresistant disease than in those with radiosensitive disease (Fig. 6C, D). Moreover, radiation led to a greater decrease in *let-7a* levels and a greater increase in IL-6 levels in radioresistant KYSE-150R cells compared with radiosensitive KYSE-150 cells (Fig S7). Furthermore, the percentage decrease in serum *let-7a* levels after 60 Gy radiotherapy was inversely correlated with tumor regression, as measured by changes in the longest tumor diameter 2 months after radiotherapy (Fig. 6E), whereas the percentage increase in serum IL-6 levels after 60 Gy radiotherapy was inversely correlated with tumor regression (Fig. 6F).

To determine whether the changes in serum *let-7a* and IL-6 levels in the early stage of radiotherapy were associated with tumor regression after radiotherapy, we collected serum samples from 27 out of the 70 patients on the 7th day after radiotherapy initiation, when patients had received 10 Gy of radiotherapy. The percentage decrease in serum *let-7a* levels and the percentage increase in serum IL-6 were inversely correlated with tumor regression (Fig. 6G, H).

## Discussion

Tumor heterogeneity drives resistance to radiotherapy since pre-existing resistant cancer cells survive and further develop, ultimately causing cancer relapse [5]. The present study revealed a more complex picture of the contribution of tumor heterogeneity to radiosensitivity. We found that mutual communication between radiosensitive and radioresistant esophageal cancer cells modulates their radiosensitivity. Specifically, radiosensitive cells increase the radiosensitivity of radioresistant cells, while radioresistant cells decrease the radiosensitivity of radiosensitive cells. Mechanistically, radiosensitive cells secrete more exosomal *let-7a* and fewer IL-6 than radioresistant cells. The exosomal *let-7a* secreted by radiosensitive cells could be taken up by radioresistant cells to increase their radiosensitivity. In contrast, IL-6 secreted by radioresistant cells decreased the radiosensitivity of radiosensitive cells. These findings have implications for cancer therapies. That is, the surgical removal of radioresistant tumor cells might increase the radiosensitivity of residual tumor cells, while the surgical removal of radiosensitive tumor cells might reduce the radiosensitivity of residual tumor cells. Moreover, the administration of a *let-7a* mimic and an anti-IL-6 antibody has the potential to modulate the communication between radioresistant and radiosensitive cells and enhance the efficacy of radiotherapy for esophageal cancer.

The identification of biomarkers that can be used to predict the response to radiotherapy and prognosis of patients with cancer might facilitate the development of a treatment plan. Although the low expression of *let-7a* in esophageal cancer tissues is associated with a poor prognosis, neither the levels of *let-7a* in esophageal cancer tissues nor in serum before radiotherapy differed significantly between patients with the radiosensitive and radioresistant disease. However, radiotherapy resulted in a more prominent decline in serum *let-7a* levels in patients with radioresistant esophageal cancer than in those with radiosensitive disease. The percentage decrease in serum *let-7a* levels at the early stage of radiotherapy was inversely associated with tumor regression after radiotherapy. Accumulating evidence has indicated that IL-6, a target of *let-7a*, confers radioresistance to different types of cancers and that serum IL-6 levels can be used to predict treatment responses and outcomes for patients with esophageal squamous cell carcinoma [25–27, 30–32]. Although neither *IL-6* mRNA levels in tumor tissues nor basal serum IL-6 levels correlated with sensitivity to radiotherapy in esophageal cancer patients, the percentage increase in serum IL-6 levels after radiotherapy was inversely correlated with tumor regression. These findings suggest that changes in the serum levels of *let-7a* and IL-6 during the early stages of radiotherapy can be used as a predictive biomarker for the response to radiotherapy.

We also found that radiation led to *let-7a* downregulation and Dicer and IL-6 upregulation. IL-6, *let-7a*, and Dicer mutually regulate each other’s expression via the following mechanisms. First, *let-7a* binds to the 3′-UTRs of Dicer and IL-6, inhibiting their expression [18, 26]. Therefore, *let-7a* downregulation increases Dicer and IL-6 expression. Second, Dicer upregulation increases the processing of pre-*let-7a* into mature *let-7a*, thereby preventing further *let-7a* downregulation following radiotherapy. Third, IL-6 triggers Dicer for proteasomal degradation [33]. As a result, IL-6 upregulation may indirectly repress pre-*let-7a* processing by downregulating Dicer expression. Finally, several miRNAs can regulate IL-6 expression [34], which suggests that Dicer may also indirectly regulate IL-6 expression via these miRNAs.

Dicer is required for DNA repair and decreased Dicer expression increases the susceptibility of cancer cells to DNA-damaging treatment, while increased Dicer expression leads to increased resistance to such treatment [19–21, 35]. Here, we found that radiation-induced less DNA damage in cells expressing higher levels of Dicer than in cells expressing lower levels of Dicer. These observations reveal that radiation-induced Dicer upregulation causes radioresistance by increasing DNA repair. Moreover, a substantial number of miRNAs are reported to positively or negatively participate in DNA repair [36]. Therefore, Dicer upregulation may affect radiosensitivity by regulating the expression of these miRNAs. Furthermore, radiotherapy induces metabolic reprogramming, which affects DNA repair and radiosensitivity [37, 38]. Dicer upregulation may affect radiosensitivity via reprogramming the metabolic status in cancer cells via different miRNAs [39–41].

In summary, we demonstrated that *let-7a*, and its target IL-6, mediate mutual communication between radiosensitive and radioresistant esophageal cancer cells, and this cell–cell communication modulates the radiosensitivity of both cell types. Moreover, we found that radiotherapy downregulates *let-7a* expression in esophageal cancer cells and that decreased *let-7a* expression leads to increased Dicer and IL-6 expression, thereby reducing the sensitivity of esophageal cancer cells to radiotherapy. Interestingly, the percentage decrease in serum *let-7a* levels and the percentage increase in serum IL-6 levels during the early stage of radiotherapy were inversely associated with tumor regression after radiotherapy, suggesting that the treatment plan for esophageal cancer can be adjusted according to alterations in serum *let-7a* and IL-6 levels during the early stage of radiotherapy.

This study has certain limitations. First, the small sample size utilized may have caused bias. Second, the serum and tissue samples were not from the same cohort of patients with esophageal cancer. In future studies, we intend to determine the expression levels of *let-7a* and IL-6 in serum and tumor tissues from the same patient cohort. We will also collect serum samples from additional esophageal cancer patients on day 7 after radiotherapy initiation and develop models to predict the radiotherapy response based on early changes in the circulating *let-7a* and IL-6 levels observed during radiotherapy.

## Methods

### Cell culture

The following human esophageal squamous cancer cell lines were procured: KYSE-150 from the Japanese Collection of Research Bioresources (Osaka, Japan); COLO680N from Deutsche Sammlung von Mikroorganismen und Zellkulturen (Braunschweig, Germany); TE15 from the Cell Resource Center for Biomedical Research (Tohoku University, Sendai, Japan); OE21 from the European Collection of Cell Cultures (Salisbury, UK); and KYSE30 from the Cell Bank, Pasteur Institute of Iran (Tehran, Iran). The radioresistant cell line KYSE-150R was established from KYSE-150 cells through fractionated irradiation [42]. All cell lines were grown in RPMI 1640 medium (Biological Industries, Kibbutz Beit Haemek, Israel) supplemented with 10% fetal bovine serum, 100 U/mL penicillin, and 100 mg/mL streptomycin. For co-culture, radiosensitivity and radioresistant cells were seeded into two separate compartments of an Ibidi culture insert (Ibidi, Martinsried, Germany) and incubated in the same medium for 48 h. The cells were cultured at 37 °C in a humidified incubator with 5% CO_2_. All cell lines were mycoplasma-free and authenticated based on polymorphic short-tandem-repeat loci after passage in our laboratory for > 6 months.

### Mouse xenograft tumor model

Female BALB/c athymic nude mice (6–8-week-old; Vital River Experimental Animal Center, Beijing, China) were randomly allocated to different groups (*n* = 5/group) and subcutaneously injected with 3 × 10^6^ cells to generate tumor xenografts. The resultant tumors were measured using calipers, and the tumor volume was calculated as follows: *V* = *L* × *W*^2^ × 0.5236 (L, long axis; W, short axis). When tumor masses became visible ( ~ 100 mm^3^), the mice received a 10 Gy dose (2 Gy/day for five consecutive days) of radiotherapy at a dose rate of 1 Gy/min and were sacrificed 21 days later. To determine the effect of *let-7a* on radiosensitivity, *let-7a* agomir or antagomir (RiboBio, Guangzhou, China) was injected intratumorally (0.5 mM/kg every three days for a total of five injections). To determine the effect of exosomes on radiosensitivity, KYSE-150 exosomes were intratumorally injected into the KYSE-150R xenografts (1 × 10^11^ particels/mice every three days for a total of five injections). To investigate the effect of IL-6 on radiosensitivity, an anti-IL-6 antibody (Affinity Bioscience, Cincinnati, OH, USA) was injected intraperitoneally (10 mg/kg/week for a total of three injections). All animal procedures were approved by the Animal Care Committee of Wenzhou Medical University.

### Patient specimens and immunohistochemical analysis

To determine the association between Dicer expression and radiosensitivity, esophageal squamous cancer tissue samples were collected from 45 patients who had received a 60 Gy dose (2 Gy/day, 5 days/week) of radiotherapy at the First Affiliated Hospital of Wenzhou Medical University (Wenzhou, China). The level of Dicer protein in tumor tissues was detected by performing immunohistochemical analysis and scored as described previously [21]. To investigate the correlation between serum levels of *let-7a* or IL-6 and radiosensitivity, we recruited another cohort of 70 patients who had esophageal squamous cancer and had received a 60 Gy dose of radiotherapy at the First Affiliated Hospital of Wenzhou Medical University. Pre-radiotherapy serum samples were collected from all 70 patients, whereas post-radiotherapy serum samples were collected from 65 patients. Additionally, we collected serum samples from 27 of the 70 patients on day 7 after the start of radiotherapy. The response to radiotherapy was evaluated as described previously [43]. The inclusion criteria for patients were as follows: (a) those who were pathologically diagnosed with esophageal cancer, (b) those who did not receive any prior systemic treatment or malignant tumor resection, and (c) those who were willing to undergo radiotherapy. Informed consent was obtained from all patients for the collection and use of clinical samples, and the study was approved by the Scientific Ethics Committee of the First Affiliated Hospital of Wenzhou Medical University.

### Radiation-associated clonogenic cell-survival assay

Cells displaying exponential growth were seeded into 6-well plates and irradiated with different doses of radiation (0, 2, 4, 6, 8, or 10 Gy) at an average rate of 100 cGy/min, followed by culture for up to 14 days. The surviving cells were stained with 0.1% Crystal Violet, and colonies containing > 50 cells were scored. The survival fraction was calculated as follows: (number of clones/number of cells plated) × plating efficiency. The plating efficiency was calculated as follows: number of clones/number of seeded unirradiated cells.

### Enzyme-linked immunosorbent assay (ELISA)

IL-6 levels in serum samples and culture medium were quantified using a human/mouse IL-6 ELISA kit (MultiSciences, Hangzhou, China) according to the manufacturer’s instructions.

### Plasmids, miRNAs, and transfection

Cells were transfected with miRNAs or plasmids using Lipofectamine 2000 (Life Technologies, Carlsbad, CA, USA) according to the manufacturer’s instructions. The intracellular *let-7a* levels were quantified 48 h after transection with miRNA mimics or inhibitors. miRNA mimics and inhibitors were purchased from RiboBio. Human Dicer knockdown and control short-hairpin RNA plasmids have been described previously [44]. The human Dicer-overexpression plasmid pDESTmycDICER was obtained from Addgene (Cambridge, MA, USA) [35].

### Comet assay

The cells were exposed to 4 Gy radiation and harvested 1 h later. Comet assays were performed as described previously [20], and comet images were visualized using a fluorescence microscope (Leica DMI3000 B; Leica, Wetzlar, Germany) and the Comet Assay Software Project (CASP, Wrocław, Poland).

### Cell proliferation

Cells were seeded into 96-well plates, and cell proliferation was assessed using the Cell Counting Kit-8 (CCK-8; MedChemExpress, Monmouth Junction, NJ, USA) according to the manufacturer’s instruction.

### Western blotting

The cells were lysed in a RIPA buffer, and the total protein was measured using the Bradford protein assay. The total cell lysate was subjected to sodium dodecyl sulfate-polyacrylamide gel electrophoresis and transferred to a polyvinylidene fluoride membrane (Merck-Millipore, Darmstadt, Germany). Blots were then incubated with primary antibodies, followed by incubation with horseradish peroxidase-conjugated secondary antibodies and detection using ECL plus reagents (GE Healthcare, Chicago, IL, USA). The primary antibodies used in this study were anti-Dicer (ab14601; Abcam, Cambridge, UK), anti-CD63 antibody (ab216130; Abcam, Cambridge, UK), anti-CD81 antibody (ab155760; Abcam, Cambridge, UK), anti-β-actin antibody (ab8227; Abcam, Cambridge, UK), anti-IL-6 (DF6087; Affinity Bioscience), and anti-glyceraldehyde 3-phosphate dehydrogenase (GAPDH; #2118; Cell Signaling Technology, Beverly, MA, USA).

### Dual-luciferase assays

Cells were co-transfected with 0.4 μg firefly luciferase reporter plasmids (including Luc-Dicer-3′-UTR1 and Luc-Dicer-3′-UTR3), 0.02 μg pRL-CMV plasmid, and 25 pmol miRNA mimics using Lipofectamine 2000 in a 24-well plate. Luciferase activity was determined 48 h after transfection using the dual-luciferase reporter assay system (Promega). Firefly luciferase activity was normalized to *Renilla* luciferase activity. The Luc-Dicer-3′-UTR1 and Luc-Dicer-3′-UTR3 plasmids have been described previously [45].

### Real-time reverse transcription-polymerase chain reaction (RT-qPCR)

Total RNA was prepared using TRIzol reagent (Life Technologies, Gaithersburg, MD, USA), and serum miRNA was extracted using a serum/plasma miRNA extraction kit (Haigene, Harbin, China). To determine the mRNA levels, RNA was reverse-transcribed using HiScript III RT SuperMix for qPCR (Vazyme Biotech Co., Ltd., Nanjing, China) according to the manufacturer’s instructions. The samples prepared in the absence of reverse transcriptase served as negative controls. RT-qPCR was performed using ChamQ Universal SYBR qPCR master mix (Vazyme Biotech Co., Ltd.) and the ABI 7500 FAST sequence detection system (Life Technologies). The primer sequences were as follows: *Dicer* (human) forward, 5′-TCCACGAGTCACAATCAACACGG-3′ and reverse, 5′-GGGTTCTGCATTTAGGAGCTAGATGAG-3′; *GAPDH* (human) forward, 5′-ATGACATCAAGAAGGTGGTG-3′ and reverse, 5′-CATACCAGGAAATGAGCTTG-3′; pri-*let-7a*-1 (human) forward, 5′-GATTCCTTTTCACCATTCACCCTGGATGTT-3′ and reverse, 5′-TTTCTATCAGACCGCCTGGATGCAGACTTT-3′; pri-*let-7a*-2 (human) forward, 5′-GCGGATCCTATGTTGTCTCTTATGAATGGCCC-3′ and reverse, 5′-CGCTCGAGATCATGATCGTTCTCACCATGTTG-3′; pri-*let-7a*-3 (human) forward, 5′-CGGAGTCCCATCGGCACCAAGACCGACTGC-3′ and reverse, 5′-TCTGTCCACCGCAGATATTACAGCCACTTC-3′; pre-*let-7a*-1 (human) forward, 5′-CACCCTGGATGTTCTCTTCACTGT-3′ and reverse, 5′-AGACCGCCTGGATGCAGACTT-3′; pre-*let-7a*-2 (human) forward, 5′-TGTTGTTTAGTGCAAGACCCAAGG-3′ and reverse, 5′-ATGCTCCCAGGTTGAGGTAGTAGG-3′; and pre-*let-7a*-3 (human) forward, 5′-CACCAAGACCGACTGCCCTTTG-3′ and reverse, 5′-ACGCTCTGTCCACCGCAGATAT-3′. To determine the miRNA levels, RNA was reverse-transcribed using an miRNA First Strand cDNA synthesis kit (by stem-loop; Vazyme Biotech Co., Ltd), and real time PCR was performed using a bulge-loop miRNA qPCR primer set (RiboBio) according to the manufacturer’s instructions. The intensity of the fluorescent dye was determined, and the level of each mRNA was normalized to that of *GAPDH*, whereas the expression of cellular miRNAs was normalized to that of *U6*, and the expression of serum miRNAs was normalized to that of spike-in cel-miR-39.

### Exosome purification, labeling, and uptake assays

Exosomes were purified using the VEX exosome isolation reagent (Vazyme Biotech Co., Ltd.) according to the manufacturer’s instructions. The exosome pellet was resuspended in 100 μL phosphate-buffered saline (PBS). For exosome-uptake experiments, KYSE-150 exosomes were labeled using a PKH67 green fluorescent cell linker kit (Sigma-Aldrich, St. Louis, MO, USA) according to the manufacturer’s instructions, added to the KYSE-150R cell medium at a concentration of 1×10^9^ particles/mL, and incubated for 24 h at 37 °C. The cells were washed with PBS, fixed with 4% paraformaldehyde, and stained with 4ʹ,6-diamidino-2-phenylindole (DAPI). Images were obtained using a Leica SP7 scanning confocal microscope (Leica Microsystems) at a magnification of 63× (NA 1.40 Oil).

### The Cancer Genome Atlas (TCGA) dataset and statistical analyses

TCGA dataset analysis was performed as described previously [46]. All experimental data were presented as the mean ± standard deviation (SD) of at least three independent experiments. Continuous variables were expressed as the mean ± SD, and categorical values were expressed as absolute and relative frequencies. The differences in continuous and categorical variables were analyzed using the Student’s *t* test or the Mann–Whitney *U* test and the chi-square test, respectively. The correlation between two variables was assessed using Spearman’s correlation analysis. Statistical analyses were performed using SPSS (v.22.0; IBM Corp., Armonk, NY, USA). All statistical tests were two-sided, and the statistical significance was set at *P* < 0.05.

### Statistical analysis

Data are presented as the mean ± standard deviation (SD) of at least three independent experiments. Statistical analyses were performed using Student’s *t* test for two groups and two-way ANOVA with Bonferroni’s post hoc test for multiple groups. Normal distribution was confirmed using the Shapiro–Wilk normality test, and homogeneity of variance was tested using Levene’s test. In all experiments, the significance level was set as α = 0.05, and *P* < 0.05 indicates significant intergroup differences. Statistical analysis was performed using GraphPad Prism 8 (GraphPad Software Inc., San Diego, CA, USA).

### Supplementary information

SupplementaryMaterials

Original western blots

## Data Availability

All the data obtained and/or analyzed during the current study were available from the corresponding authors on reasonable request.
