# Supplementary material for: Mutual communication between radiosensitive and radioresistant esophageal cancer cells modulates their radiosensitivity
Source: Cell Death Dis. 2023 Dec 19;14(12):846. doi: 10.1038/s41419-023-06307-9 (PMC10730729; doi:10.1038/s41419-023-06307-9)
Supplement: Supplementary file 1 — SupplementaryMaterials [file 41419_2023_6307_MOESM1_ESM.docx]

**Supplementary Materials**


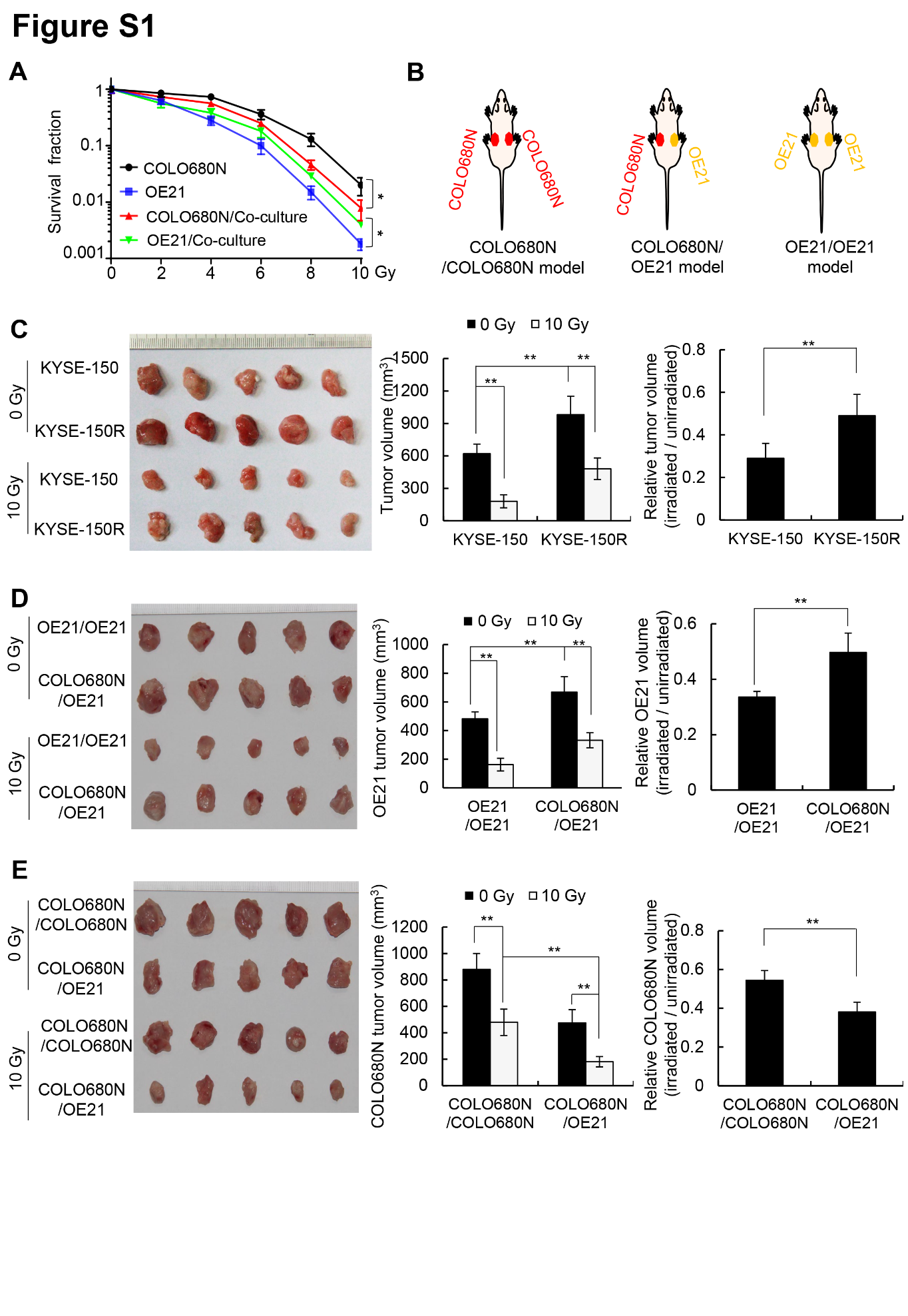


**Figure S1. Cell–cell communication between radiosensitive and radioresistant esophageal cancer cells.** **(A)** The radiosensitive OE21 cells alone, the radioresistant COLO680N cells alone, the OE21 cells co-cultured with COLO680N cells for 48 h, and the COLO680N cells co-cultured with OE21 cells for 48 h were exposed to different doses of radiation. Subsequent cell survival was determined using clonogenic assays. Data are expressed as the mean ± SD of the values from three independent experiments. **P* < 0.05 (two-sided Student’s *t*-test). **(B)** Schematic displaying the establishment of the radioresistant (COLO680N/COLO680N) tumor xenograft mouse model (left), the radioresistant/radiosensitive (COLO680N/OE21) xenograft mouse model (middle), and the radiosensitive (OE21/OE21) xenograft mouse model (right). **(C)** Subcutaneous xenografts of either KYSE-150 or KYSE-150R cells were established in athymic nude mice and then subjected to 10 Gy of radiation. Xenograft tumors were imaged (left), the average volume was determined (middle), and the volumes of irradiated tumors relative to those of unirradiated tumors were calculated (right). **(D)** OE21 xenografts on the right flank of the OE21/OE21 mouse model and COLO680N/OE21 mouse model were either exposed to 10 Gy of irradiation or left unirradiated. Xenograft tumors were photographed (left), the average volume was determined (middle), and the volumes of irradiated xenografts relative to those of unirradiated xenografts were calculated (right). **(E)** COLO680N xenografts on the left flank of the COLO680N/COLO680N mouse model and the COLO680N/OE21 mouse model were either exposed to 10 Gy of radiation or left unirradiated. Xenograft tumors were photographed (left), the average volume was determined (middle), and the volumes of irradiated xenografts relative to those of unirradiated xenografts were calculated (right). Data (C-E) are expressed as the mean ± SD of the values obtained from five xenografts. ***P* < 0.01 (two-sided Student’s *t*-test).


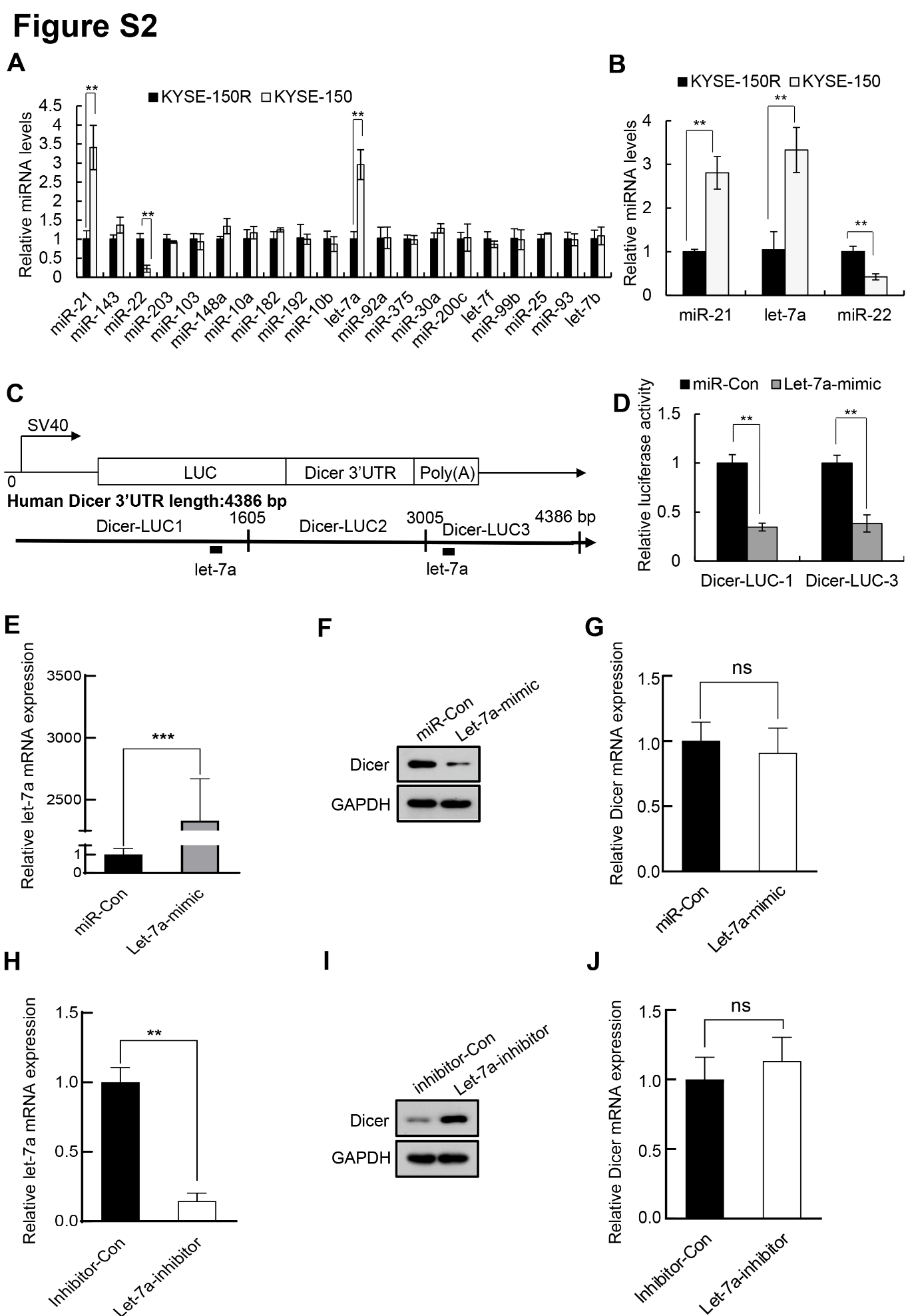


**Figure S2. *Let-7a* is upregulated and decreases Dicer expression in radiosensitive esophageal cancer cells.** **(A)** Quantification of miRNA expression in the culture medium of KYSE-150 and KYSE-150R cells. **(B)** Quantification of intracellular *miR-21, let-7a*, and *miR-22* levels in KYSE-150R cells and KYSE-150 cells. **(C)** Bioinformatic analysis of the binding sites of *let-7a* in the 3′-UTR region of Dicer mRNA. **(D)** Luciferase activities of Dicer 3′-UTR reporters in KYSE-150 cells transfected with a *let-7a* or control mimic. **(E-G)** KYSE-150R cells were transfected with either a *let-7a* or control mimic. The levels of *let-7a* (E), and Dicer protein (F), and mRNA (G) were determined 48 h after transfection. **(H-J)** KYSE-150 cells were transfected with either a *let-7a* or control inhibitor. The levels of *let-7a* (H) and Dicer protein (I) and mRNA (J) were determined 48 h after transfection. Data (A, B, D, E, G, H, and J) are expressed as the mean ± SD of the values from three biological replicates. ****P* < 0.001, ***P* < 0.01, ns, not significant (two-sided Student’s *t*-test).


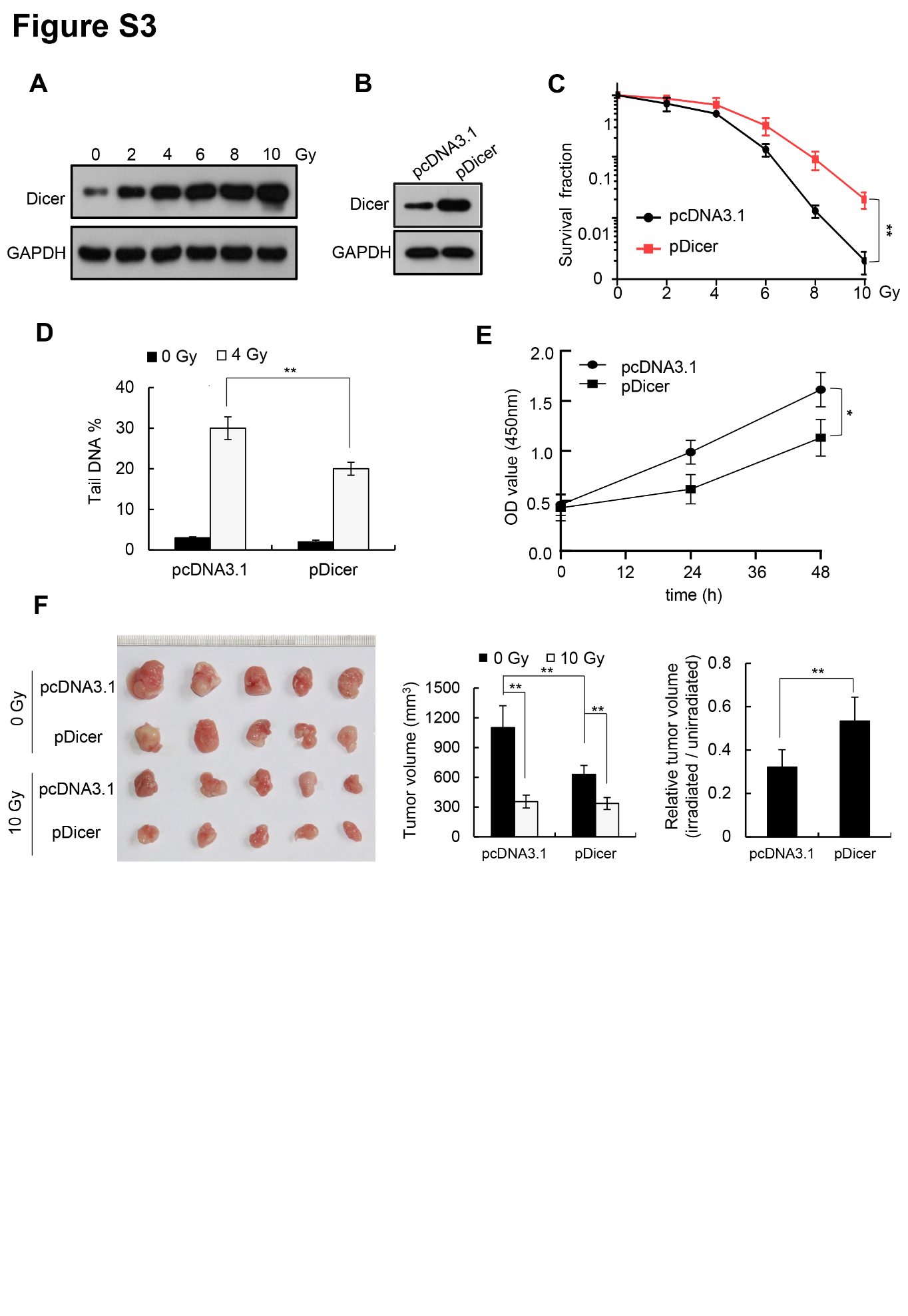


**Figure S3. Dicer overexpression decreases the sensitivity of esophageal cancer cells to radiotherapy.** **(A)** Representative western blotting images of Dicer expression in KYSE-150 cells exposed to different doses of X-ray radiation. **(B)** Representative western blotting images of Dicer expression in control and Dicer-overexpressing KYSE-150 cells. **(C)** Control and Dicer-overexpressing KYSE-150 cells were subjected to different doses of irradiation, and cell survival was determined using clonogenic assays. **(D)** Control and Dicer-overexpressing KYSE-150 cells were subjected to 4 Gy of irradiation, and DNA breaks were determined using comet assays 1 h after irradiation. **(E)** The proliferation of control and Dicer-overexpressing KYSE-150 cells was determined using CCK-8 assays. Data (C-E) are expressed as the mean ± SD of the values from three biological replicates. ***P* < 0.01 (two-sided Student’s *t*-test), **P* < 0.05. **(F)** Subcutaneous xenografts of either Dicer-overexpressing or control KYSE-150 cells were established in athymic nude mice and then subjected to 10 Gy of irradiation. Xenograft tumors were photographed (left), the average volume was determined (middle), and the volumes of irradiated tumors relative to those of unirradiated tumors were calculated (right). Data are expressed as the mean ± SD of the values obtained from five xenografts.***P* < 0.01(two-sided Student’s *t*-test).


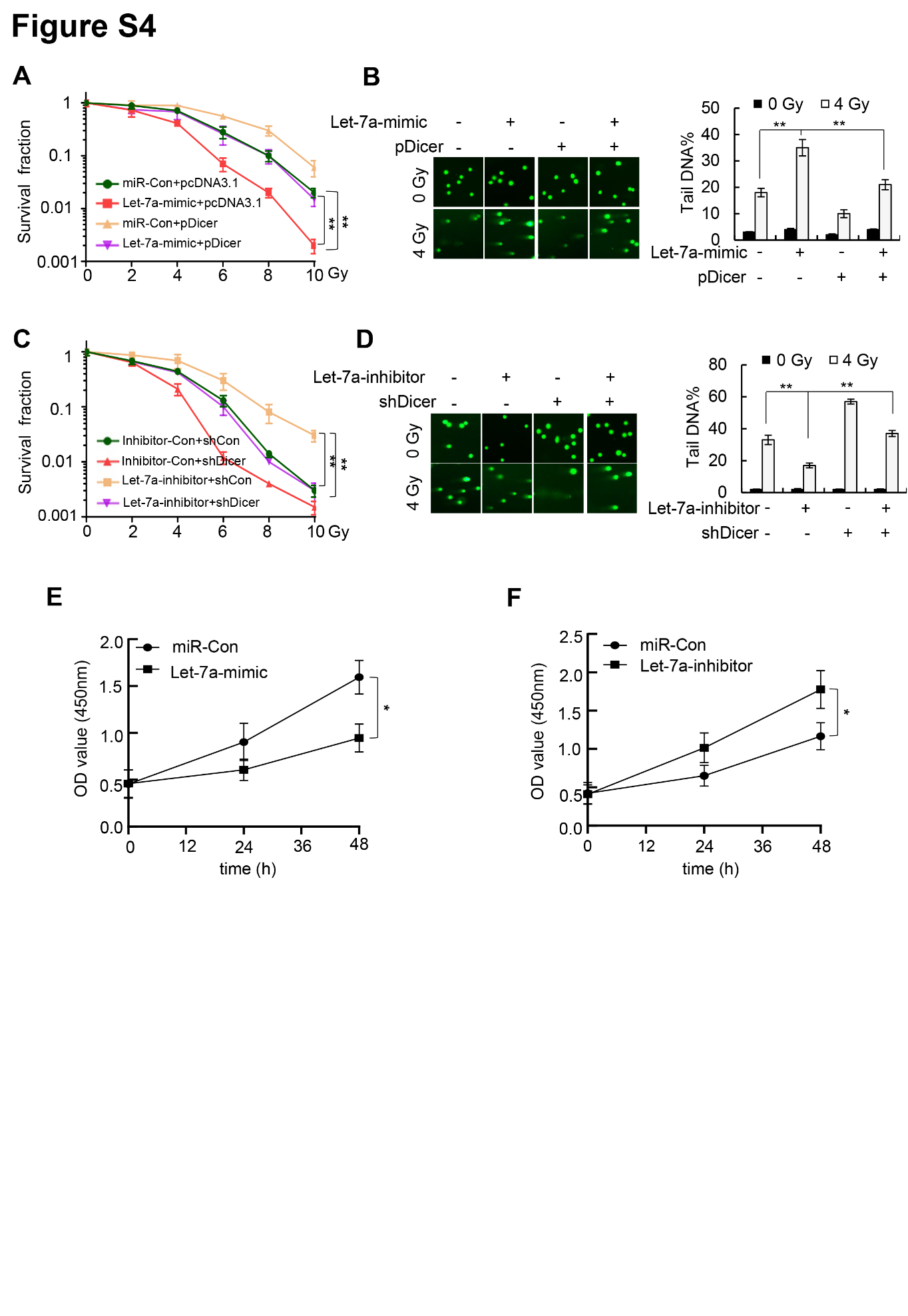


**Figure S4. *Let-7a* increases the sensitivity of esophageal cancer cells to radiotherapy by regulating Dicer expression.** **(A, B)** COLO680N cells were co-transfected with a miR-Con/*let-7a*-mimic and pcDNA3.1/pDicer, as indicated, and treated with different doses of radiation 48 h after transfection. Cell survival was determined using clonogenic assays (A), and DNA breaks were measured using comet assays (B). **(C, D)** OE21 cells were co-transfected with an inhibitor-Con/*let-7a*-inhibitor and shCon/shDicer, as indicated, and treated with different doses of radiation 48 h after transfection. Cell survival was determined using clonogenic assays (C), and DNA breaks were measured using comet assays (D). **(E)** KYSE-150R cells were transfected with either a *let-7a* or control mimic, and cell proliferation was determined using CCK-8 assays. **(F)** KYSE-150 cells were transfected with either a *let-7a* or control inhibitor, and cell proliferation was determined using CCK-8 assays. Data (A-F) are expressed as the mean ± SD of the values from three independent experiments. ***P* < 0.01, **P* < 0.05 (two-sided Student’s *t*-test).


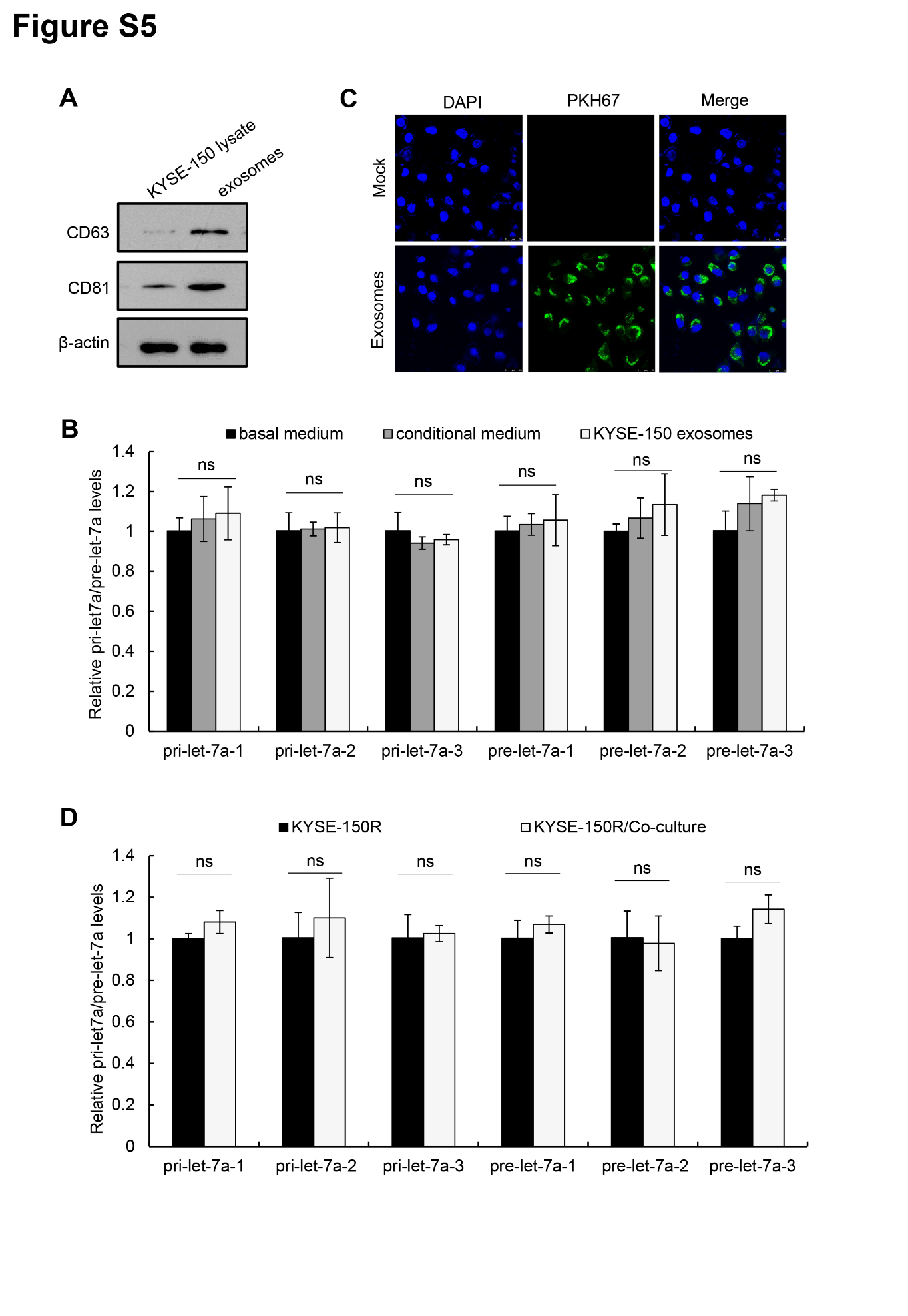


**Figure S5. KYSE-150 exosome uptake by KYSE-150R cells does not affect intracellular *pri-let-7a* and *pre-let-7a* levels.** **(A)** Representative western blotting images of CD63 and CD81 proteins in the isolated exosomes and cell lysate from KYSE-150 cells. **(B)** KYSE-150R cells were incubated for 48 h with either basal medium, conditioned medium collected from KYSE-150 cells, or basal medium supplemented with KYSE-150 exosomes. The intracellular *pri-let-7a* and *pre-let-7a* levels were then quantified using real-time RT-PCR. **(C)** Images of KYSE-150R cells were taken using a confocal microscope after incubation with PKH67-labeled (green) KYSE-150 exosomes for 24 h, followed by fixing and staining with DAPI (blue). **(D)** Intracellular *pri-let-7a* and *pre-let-7a* levels in KYSE-150R cells were quantified using real-time RT-PCR after being co-cultured with KYSE-150 cells for 48 h. Data (B and D) are expressed as the mean ± SD of values from three independent experiments. Ns, not significant (two-sided Student’s *t*-test).


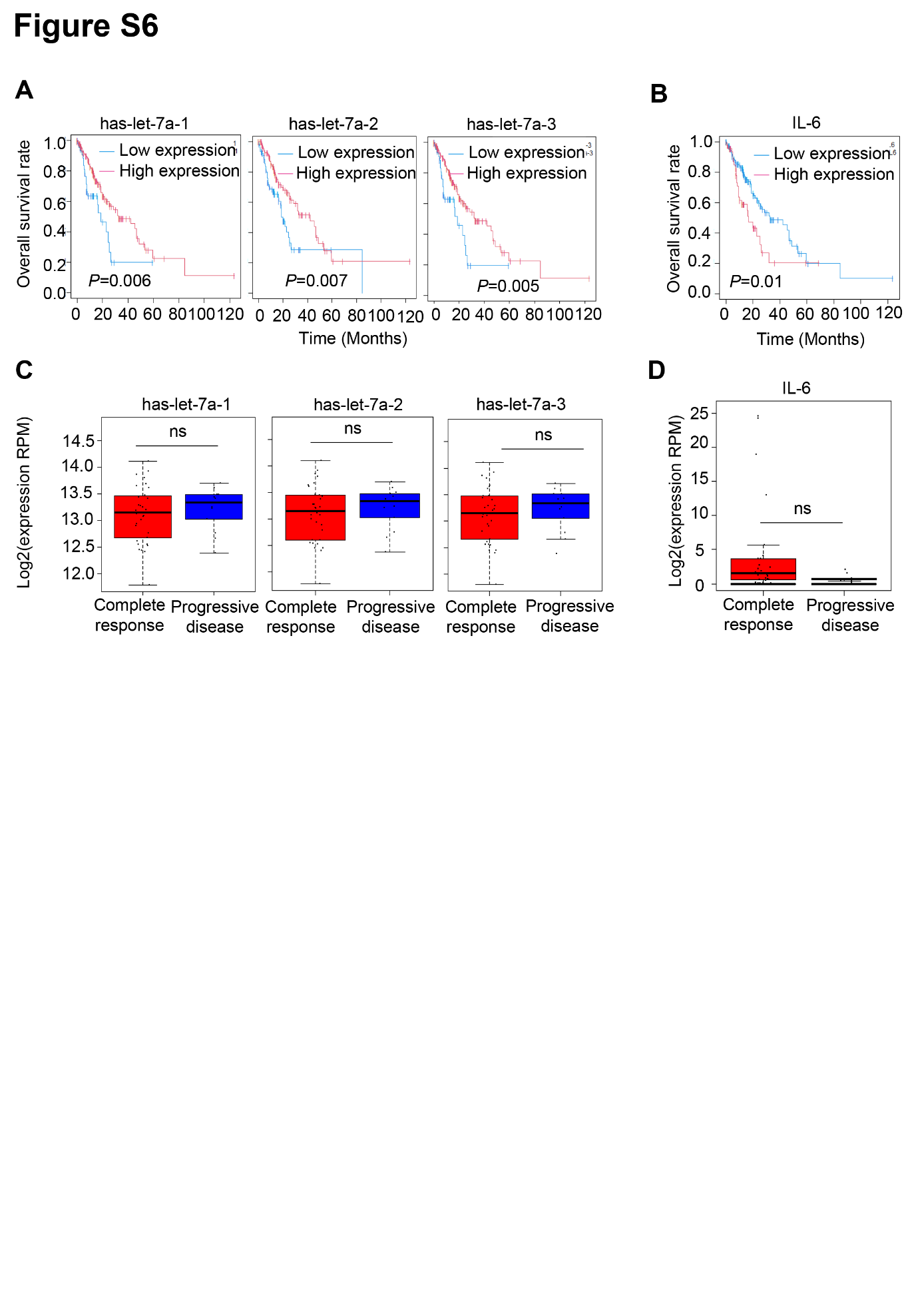


**Figure S6.** **Correlation of *let-7a* and *IL-6* expression in esophageal cancer tissues with clinical parameters. (A, B)** Kaplan–Meier survival curves for esophageal cancer patients according to *let-7a* (A) and *IL-6* mRNA (B) expression levels in tumor tissues. **(C, D)** Expression levels of *let-7a* (C) and *IL-6* mRNA (D) in patients exhibiting a complete response and progressive disease. Data (A–D) were downloaded from TCGA. Ns, not significant (two-sided Student’s *t*-test).


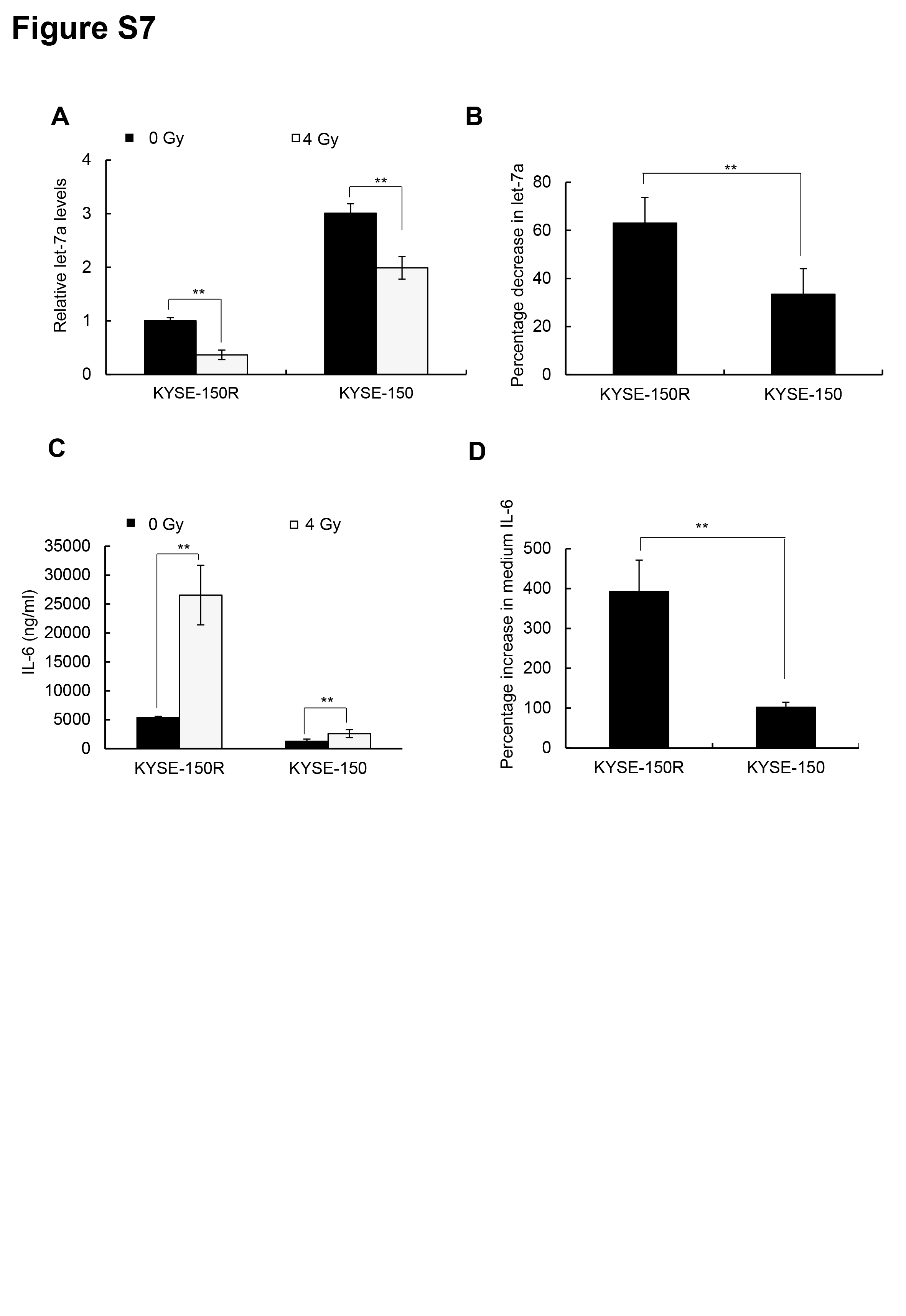


**Figure S7. Radiation results in a greater decrease in *let-7a* and increase in IL-6 in radioresistant KYSE-150R cells than in radiosensitive KYSE-150 cells.** **(A)** Levels of *let-7a* in KYSE-150 and KYSE-150R cells either irradiated with 4 Gy or left unirradiated. **(B)** The percentage decrease in *let-7a* levels was determined after treatment with 4 Gy of radiation in KYSE-150 and KYSE-150R cells. The percentage decrease in *let-7a* levels was calculated as follows: (unirradiated cell *let-7a* level − irradiated cell *let-7a* level)/unirradiated cell *let-7a* level × 100%. **(C)** Levels of IL-6 in culture medium of KYSE-150 and KYSE-150R cells either irradiated with 4 Gy or left unirradiated. **(D)** The percentage increase in IL-6 levels was determined after treatment with 4 Gy of radiation in KYSE-150 and KYSE-150R cells. The percentage increase in IL-6 levels was calculated as follows: (irradiated cell IL-6 level − unirradiated cell IL-6 level)/unirradiated cell IL-6 level × 100%. Data (A-D) are expressed as the mean ± SD of values from three independent experiments. ***P* < 0.01 (two-sided Student’s *t*-test).

**Table S1.** Top 20 miRNAs with the highest expression in esophageal cancer.

| miRNAs | Expression |
| --- | --- |
| hsa-mir-21 | 338192 |
| hsa-mir-143 | 139155.1 |
| hsa-mir-22 | 63249.62 |
| hsa-mir-203 | 45289.49 |
| hsa-mir-103-1 | 27962.5 |
| hsa-mir-148a | 24799.07 |
| hsa-mir-10a | 22647.99 |
| hsa-mir-182 | 20127.97 |
| hsa-mir-192 | 19965.24 |
| hsa-mir-10b | 18542.06 |
| hsa-let-7a-2 | 14965.59 |
| hsa-mir-92a-2 | 14812.66 |
| hsa-mir-375 | 14608.27 |
| hsa-mir-30a | 13693.85 |
| hsa-mir-200c | 13492.98 |
| hsa-let-7f-2 | 13394.93 |
| hsa-mir-99b | 12184.82 |
| hsa-mir-25 | 11998.52 |
| hsa-mir-93 | 10805.26 |
| hsa-let-7b | 10293.72 |
