# Supplementary material for: Mutual communication between radiosensitive and radioresistant esophageal cancer cells modulates their radiosensitivity
Source: Cell Death Dis. 2023 Dec 19;14(12):846. doi: 10.1038/s41419-023-06307-9 (PMC10730729; doi:10.1038/s41419-023-06307-9)

Full and uncropped western blots

Figure 2A

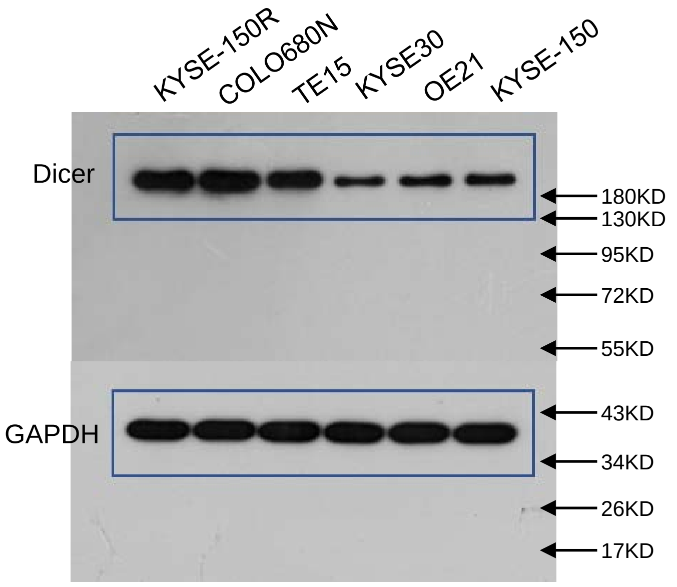

Figure 2B

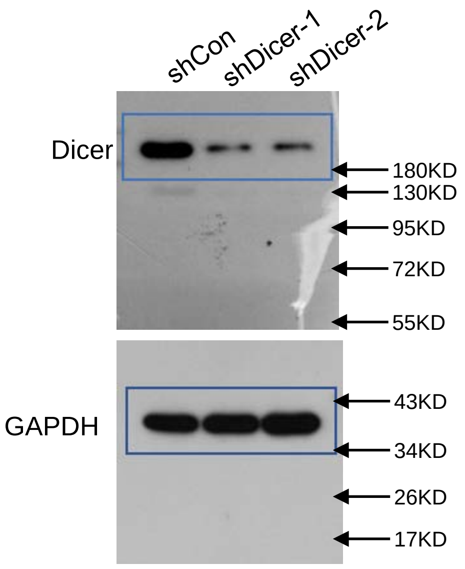

Figure 4F

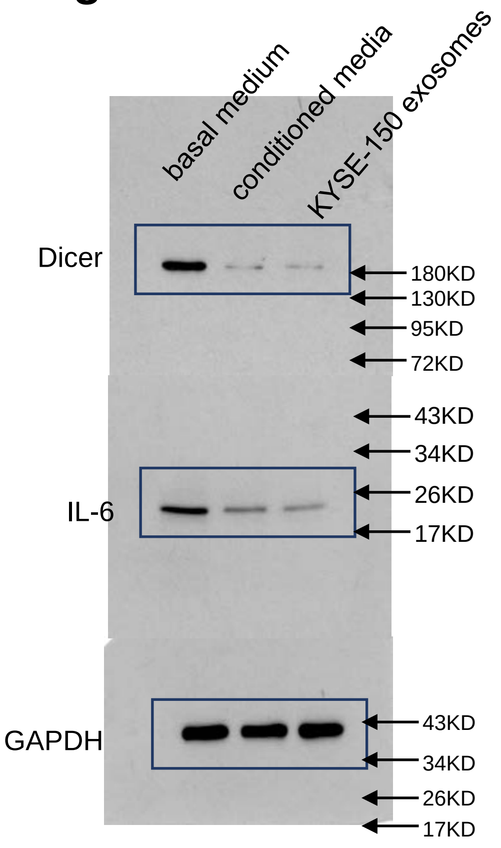

Figure S2F

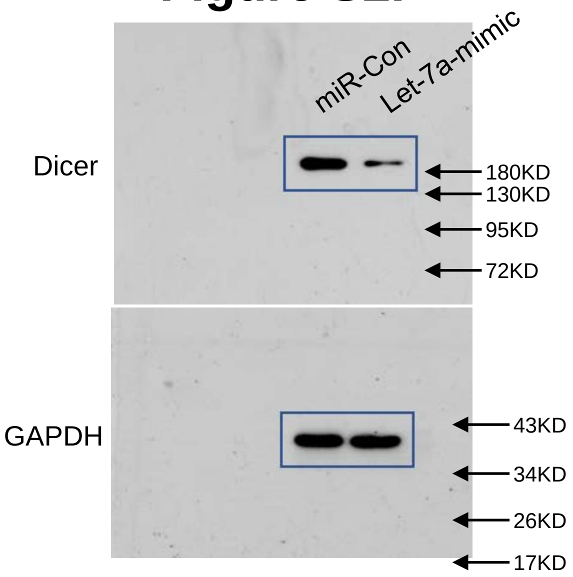

Figure S2I

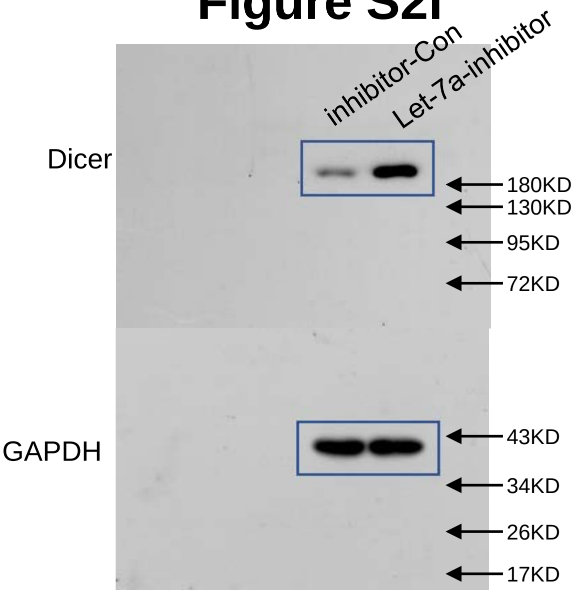

Full and uncropped western blots

Figure 5A

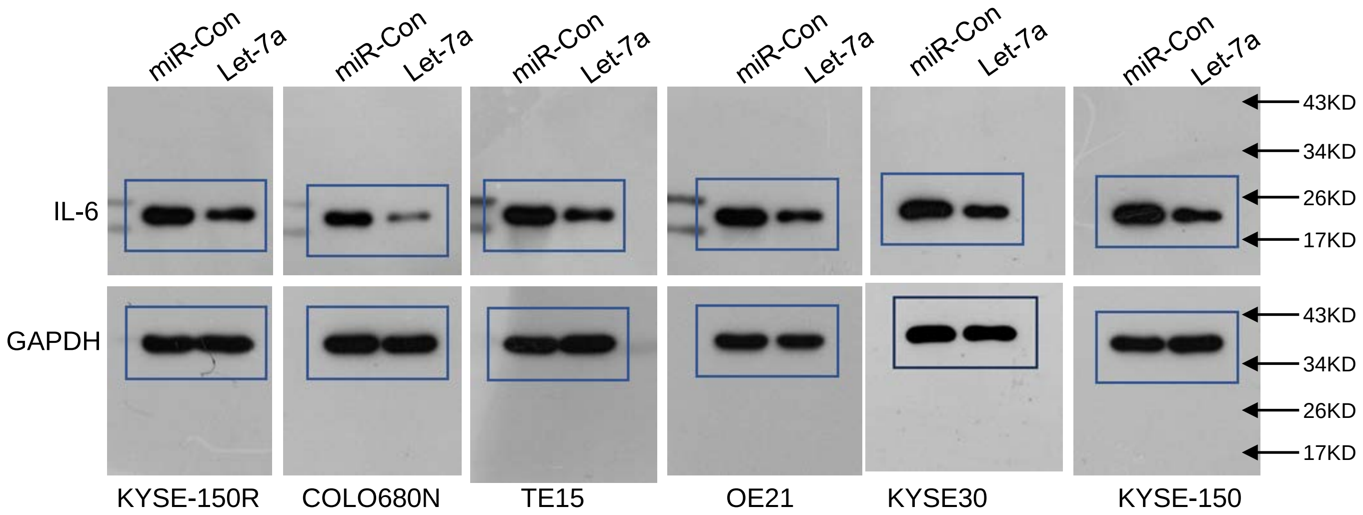

Figure S3A

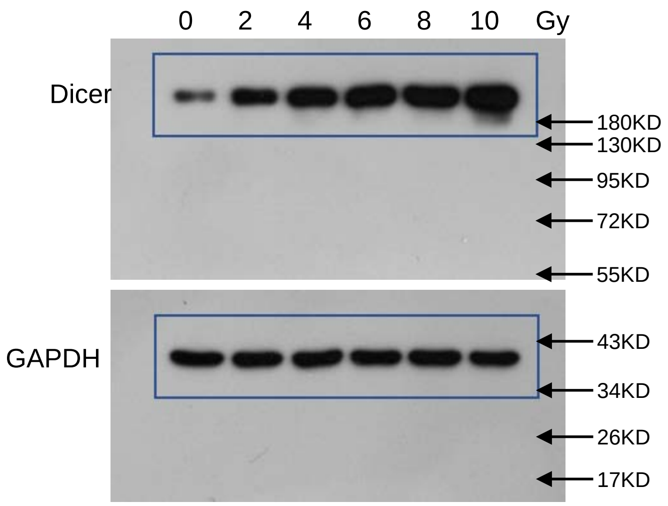

Figure S3B

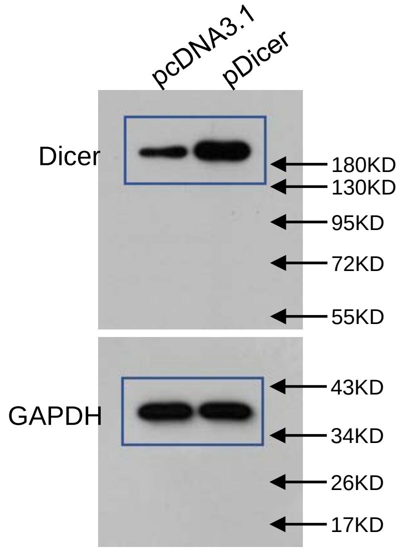

Figure S5A

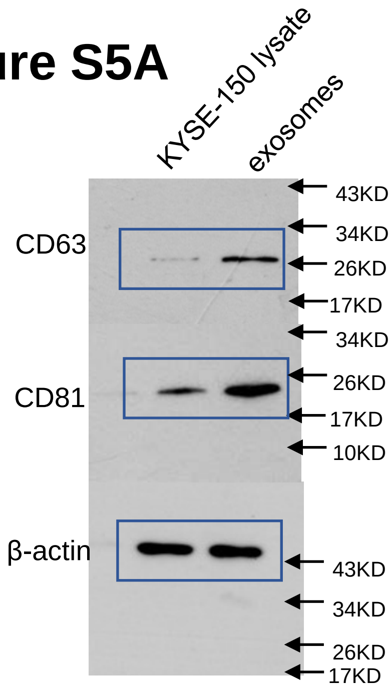

the western blot shown in cover letter

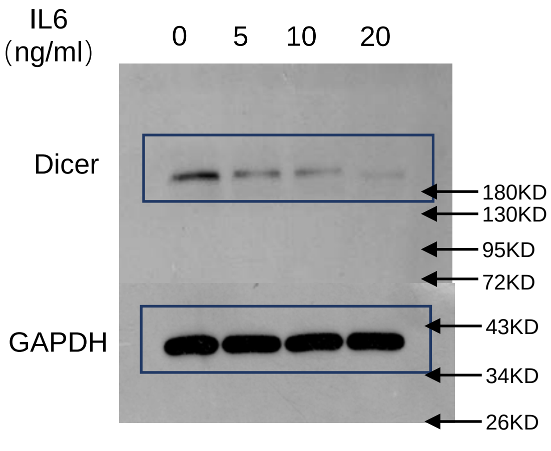

Supplement: Supplementary file 2 — Original western blots [file 41419_2023_6307_MOESM2_ESM.pdf]
